# Cytotoxic Natural Products from *Cryptomeria japonica* (Thunb. ex L.) D.Don

**DOI:** 10.3390/ijms252413735

**Published:** 2024-12-23

**Authors:** Bjørn Tobiassen Heieren, Anja Strandvoll Dyrdal, Lars Herfindal, Bjarte Holmelid, Cato Brede, Heidi Lie Andersen, Torgils Fossen

**Affiliations:** 1 Department of Chemistry and Centre for Pharmacy, University of Bergen, N-5007 Bergen, Norway; bjorn.t.heieren@gmail.com (B.T.H.); bjarte.holmelid@uib.no (B.H.); 2 Department of Clinical Science and Centre for Pharmacy, University of Bergen, N-5009 Bergen, Norway; anja.dyrdal@student.uib.no (A.S.D.); lars.herfindal@uib.no (L.H.); 3 Department of Medical Biochemistry, Stavanger University Hospital, N-4011 Stavanger, Norway; cato.brede@uis.no; 4 University Gardens, University of Bergen, Allégt. 41, N-5007 Bergen, Norway; heidi.andersen@uib.no

**Keywords:** *Cryptomeria japonica*, hinokiflavone 7″-O-β-glucopyranoside, cytotoxic activity, 2D NMR

## Abstract

*Cryptomeria japonica* is a commercially important tree native to Japan. The tree belongs to the ancient genus *Cryptomeria* and has found important uses as a medicinal plant, as well as a main source of timber in Japan. In recent years, there has been an increased interest in discovering extended uses of *C. japonica* as a source of novel bioactive natural products with potential applications as lead compounds for active principles of future drugs. The compounds were isolated by a combination of two-phase extraction, XAD-7 Amberlite column chromatography, Sephadex LH-20 column chromatography and preparative High Performance Liquid Chromatography (HPLC). The structures were determined by a combination of several 1D and 2D Nuclear Magnetic Resonance (NMR) experiments and high-resolution mass spectrometry. Here, we report on the isolation and characterization of the novel biflavone glucoside hinokiflavone 7″-*O*-β-glucopyranoside, in addition to sixteen known compounds including the flavonols quercetin, quercetin 3-*O*-α-rhamnopyranoside and quercetin 3-*O*-β-galactopyranoside, the dihydroflavonols taxifolin 3-*O*-β-glucopyranoside, taxifolin 7-*O*-β-glucopyranoside, the flavanones naringenin, naringenin 7-*O*-β-galactopyranoside and eriodictyol 4′-*O*-β-glucopyranoside, the flavanol catechin, the biflavonoid amentoflavone, the dihydrochalcone phloretin 2′-*O*-β-glucopyranoside, the sesquiterpenoid roseoside, the polyphenolic compounds chlorogenic acid, methyl chlorogenate and the flavanocoumarins catechin-(7,8)-7″-(3,4 dihydroxyphenyl)-dihydro-8″(3H)-pyranone, and mururin A. The compounds exhibited low-to-moderate cytotoxic activity against MOLM-13 leukemia cells.

## 1. Introduction

**Figure 1:**
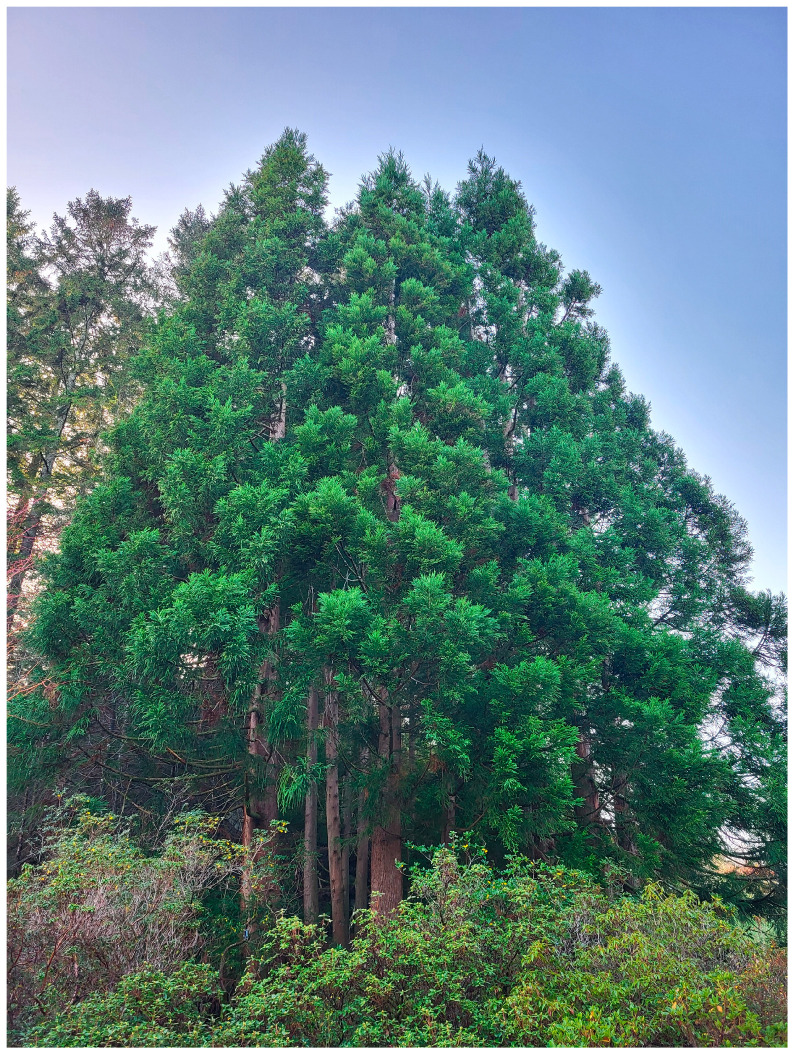
*Cryptomeria japonica* grown in the Arboretum of University of Bergen. Photo: Heidi Lie Andersen. Photo was taken on 14 November 2024.

*Cryptomeria* is an ancient genus of evergreen trees which have existed since the Late Eocene (38–33.9 million years ago) [1]. The only currently living species of this genus is *Cryptomeria japonica* (Thunb. ex L.) D.Don (Figure 1), which is the national tree of Japan [1]. Fossils from species of *Cryptomeria* have been found on the Eurasian continent from the Tertiary period. The oldest fossil that can be identified to *Cryptomeria* is a fossil of a cone from the Paleocene period, the oldest period of the Tertiary period ([2], p. 52). First and foremost, the tree has been used as a building material but also has several applications in traditional medicine in Asia for the treatment of liver problems and stomach ulcers and as cough medicine [3]. More recently, studies have been conducted in which compounds from the tree have shown biological activity against cancer [4,5,6,7], bacteria [8], and fungi [9,10,11], in addition to exhibiting anti-inflammatory activity [7,12].

Ho et al. (2015) conducted a study in which the leaf extract of Japanese cedar was evaluated as a potential agent against lung cancer [4]. The compound ferruginol is present in significant amounts in Japanese cedar and was isolated from the extract and used further in in vitro and in vivo experiments against non-small-cell lung cancer. The diterpenoid ferruginol demonstrated high biological activity against this type of lung cancer, which accounts for approximately 85% of all cases of lung cancers. The anticancer effect of ferruginol is correlated with its activation of the cell death process of two of the cell lines that cause small-cell lung cancer [4]. The terpene cryptotrione has been isolated from the bark of *C. japonica* and tested by Chen et al. (2010) against oral cancer. The compound showed significant cytotoxicity, although to a somewhat lesser extent than the clinically used anticancer agent, etoposide [5]. The diterpene 6-hydroxy-5,6-dehydrosugiol isolated from the bark of *C. japonica* is a compound with significant anticancer activity against prostate cancer and is considered as an active principle of potential future anticancer drugs [6]. The monoterpenes terpinen-4-ol, α-pinene, α-terpineol, and sabinene are often found in aromatic plants and have shown both anticancer and anti-inflammatory activity [7]. Shyur et al. (2008) reported that the terpenes sugiol, 12-hydroxy-6,7-secoabieta-8,11,13-triene-6,7-dial, and (1S,6R)-2,7(14),10-bisabolatrien-1-ol-4-one isolated from the wood of *C. japonica* exhibited significant anti-inflammatory activity [7]. Shyur et al. (2011) demonstrated that (1S,6R)-2,7(14),10-bisabolatrien-1-ol-4-one isolated from *C. japonica* is a potent stimulant for the production of heme oxygenase-1 (which prevents vascular inflammation) and for inhibiting the proteins TNF-α, COX-2, iNOS, and the signaling molecule IL-6, which all are involved in the process of inflammation. These properties contribute to liver protection and confirm the traditional medicinal observation that Japanese cedar is active against liver damage [12]. Among antibacterial compounds, ferruginol showed high activity, as did isopimaric acid, sugiol, and sandaracopimarinol [8]. Several compounds isolated from *C. japonica* exhibit significant antifungal properties. These include the diterpenoid 7,11,14-trioxoabieta-8,12-diene (cryptoquinone) isolated from the bark and the sequiterpenoids epi-cubenol and δ-cadinene isolated from wood chips derived from *C. japonica* [9,10,11]. Oil from leaves and bark of *C. japonica* have also shown significant activity against yellow fever mosquito larvae [13]. In excellent correlation with the fact that *C. japonica* is a unique species without any close relatives, an unusually high number of novel natural products have been identified from this species. A substantial number of high-quality research papers have been published with respect to the characterization of natural products from *C. japonica*. The fact that no extensive review of natural products from this species exist in the current literature encouraged us to provide a systematized and comprehensive overview of the natural products identified from *C. japonica* (Table 1, Table 2, Table 3, Table 4, Table 5, Table 6 and Table 7). In total, 359 different natural products have been reported from *C. japonica* in the current literature (Table 1, Table 2, Table 3, Table 4, Table 5, Table 6 and Table 7) [14,15,16,17,18,19,20,21,22,23,24,25,26,27,28,29,30,31,32,33,34,35,36,37,38,39,40,41,42,43], including 33 novel compounds. These novel compounds have previously been isolated from the bark, needles, and heartwood of *C. japonica*. The majority of the novel compounds reported from *C. japonica* in the current literature are sesquiterpenoids [24,26,30] including elem-1-en-4,11-diol, 11-acetoxyeudesman-4α-ol, eudesmane-5α,11-diol, 3-eudesmene-1β,11-diol, 1β-acetoxy-3-eudesmen-11-ol, 4-eudesmene-1β,11-diol, 1β-acetoxy-4-eudesmen-11-ol, 7-epi-γ-eudesmol, 7-epi-4-eudesmene-1β,11-diol, 1β-acetoxy-4(15)-eudesmen-11-ol [24], (4S)-2,6,10-bisaboratrien-4-ol-1-one, 1,8-epoxy-1(6),2,4,7,10-bisaborapenta-en-4-ol, and 1-methoxy-4-cadinene [26], in addition to ferrugicadinol A and ferrugicryptomeridiol [30] and diterpenoids including the abietane-type diterpenoids, 7α-butoxyabieta-8,12-diene-11,14-dione, 6α,7α-dihydroxyabieta-8,12-dien-11,14-dione, and 6α,7β-dihydroxyabieta-8,12-diene-11,14-dione [28], as well as 6,12-dihydroxyabieta-5,8,11,13-tetraen-7-one, 6β-hydroxyferruginol, 7α,8α-epoxy-6α-hydroxyabieta-9(11), 13-dien-12-one, (5*R*,10*S*)-12-methoxyabieta-6,8,11,13-tetraene, *ent*-kaur-15-en-17-al, and (+)-16-acetylkaurane-16,17-diol [31], in addition to 8,13-dioxo-14,15,17-trinorlabdan-19-oic acid, 12-hydroxy-11-methoxyabieta-8,11,13-trien-7-one, 6α,11-dihydroxy-12-methoxyabieta-8,11,13-trien-7-one, 6,12-dihydroxy-11-methoxyabieta-5,8,11,13-tetraen-7-one, an acetal formed by abieta-8,11,13-triene-6α,7α,11,12-tetraol, and imbricataloic acid, a self-condensation product of imbricataloic acid, as well as an acetal formed by abieta-8,11,13-triene-6α,7β,12-triol and 12-hydroxy-6,7-secoabieta-8,11,13-triene-6,7-dial [32]. However, only a limited number of aromatic compounds have been characterized from *C. japonica*. Su et al. identified eight known flavonoids and 10 lignans from the leaves of *C. japonica* including the 2 novel lignans *cis*-dihydrodehydrodiconiferyl alcohol triacetate and *seco*dihydrodehydrodiconiferyl alcohol tetraacetate [35]. The flavonoids hitherto identified from *C. japonica* have been restricted to monomeric or dimeric flavonoid aglycones [37], and no flavonoid glycosides nor any novel flavonoids have hitherto been identified from this species. Flavonoids have been associated with multiple positive health effects including anti-inflammatory and antiviral activity [43,44]. Several individual flavonoids exhibit selective cytotoxic activity against cancer cells; however, their biological activity strongly depends on their individual molecular structures [45,46]. Structural isomers of flavonoid phenolic compounds, such as isocoumarins, are widely recognized for their diverse biological activities, including antioxidant, antimicrobial, and anticancer properties [47,48]. In this paper, we report on the isolation and structure elucidation of seventeen natural products including a novel dimeric flavonoid glucoside. The cytotoxicity of selected compounds against MOLM-13 leukemia cells is also revealed. For the first time, a comprehensive and systematic overview of natural products characterized from *C. japonica* is provided (Table 1, Table 2, Table 3, Table 4, Table 5, Table 6 and Table 7).

## 2. Results

**Figure 2:**
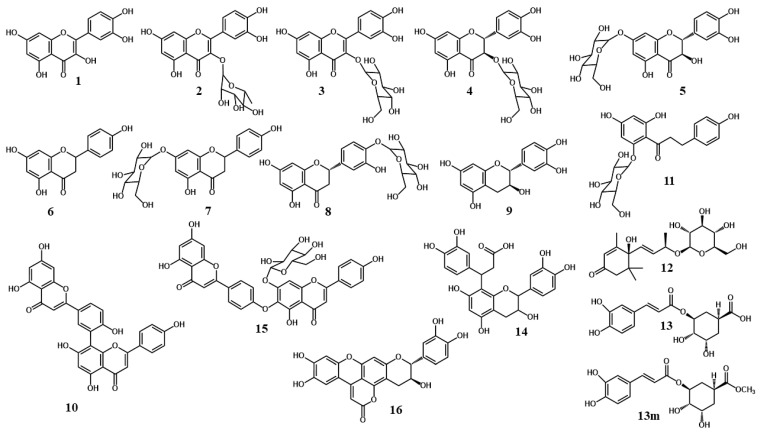
Molecular structures of quercetin (**1**), quercetin 3-*O*-*α*-rhamnopyranoside (**2**), quercetin 3-*O*-*β*-galactopyranoside (**3**), taxifolin 3-*O*-*β*-glucopyranoside (**4**), taxifolin 7-*O*-*β*-glucopyranoside (**5**), naringenin (**6**), naringenin 7-*O*-*β*-galactopyranoside (**7**), eriodictyol 4′-*O*-*β*-glucopyranoside (**8**), catechin (**9**), amentoflavone (**10**), phloretin 2′-*O*-*β*-glucopyranoside (**11**), roseoside (**12**), chlorogenic acid (**13**), and methyl chlorogenate (**13m**), in addition to the rare natural products catechin-(7,8)-7″-(3,4-dihydroxyphenyl)-dihydro-8″(3H)-pyranone (**14**), hinokiflavone 7″-*O*-*β*-glucopyranoside (**15**), and Mururin A (**16**).

Seventeen natural products were isolated from the concentrated methanolic extract of leaves of *Cryptomeria japonica* by using two-phase extraction against petroleum ether and ethyl acetat, followed by XAD-7 column chromatography, Sephadex LH-20 column chromatography, and preparative High Performance Liquid Chromatography (HPLC). Following this procedure, 17 natural products were characterized. The molecular structures of the 16 known natural products quercetin (**1**), quercetin 3-*O*-*α*-rhamnopyranoside (**2**), quercetin 3-*O*-*β*-galactopyranoside (**3**), taxifolin 3-*O*-*β*-glucopyranoside (**4**), taxifolin 7-*O*-*β*-glucopyranoside (**5**), naringenin (**6**), naringenin 7-*O*-*β*-galactopyranoside (**7**), eriodictyol 4′-*O*-*β*-glucopyranoside (**8**), catechin (**9**), amentoflavone (**10**), phloretin 2′-*O*-*β*-glucopyranoside (**11**), roseoside (**12**), chlorogenic acid (**13**), methyl chlorogenate (**13m**), in addition to the rare natural products catechin-(7,8)-7″-(3,4-dihydroxyphenyl)-dihydro-8″(3H)-pyranone (**14**), and Mururin A (**16**) (Figure 2) were determined by using a combination of several 1D and 2D Nuclear Magnetic Resonace (NMR) spectroscopic techniques and High-Resolution Mass Spectrometry (HR-MS) (Tables S1–S15).

**Figure 3:**
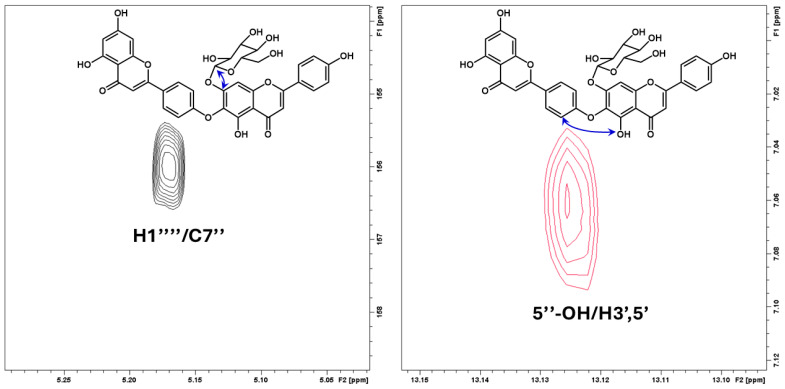
Expanded regions of the 2D ^1^H-^13^C HMBC spectrum (**left**) and the 2D ^1^H-^1^H ROESY spectrum (**right**) of hinokiflavone 7″-*O*-*β*-glucopyranoside (**15**) showing important crosspeaks for determination of linkages between the substructures of the compound. Blue arrows highlight the observed correlations in the molecular structure.

The downfield region of the 1D 1H NMR spectrum of compound **15** (Figure S1) showed a 4H AA’XX’ system at ẟ 8.00 (‘d’ 9.0, H2′/6′) and ẟ 7.06 (‘d’ 9.0, H3′/5′), a 2H AB system at ẟ 6.49 (d 2.1, H8) and ẟ 6.20 (d 2.1, H6), and a 1H singlet at ẟ 6.86 (H3), in addition to a further 4H AA’XX’ system at ẟ 7.99 (‘d’ 8.9, H2‴/6‴) and ẟ 6.96 (‘d’ 8.9, H3‴/5‴) and 2 1H singlets at ẟ 6.93 (H3″) and ẟ 7.19 (H8), which is in accordance with the biflavonoid aglycone 4′,6″-O-biapigenin (hinokiflavone). The identity of hinokiflavone aglycone was further supported by the 30 ^13^C resonances belonging to the aglycone which were identified and completely assigned by the combined information in the 2D ^1^H-^13^C HMBC spectrum (Figure S3) and the 2D ^1^H-^13^C HSQC spectrum (Figure S4) of **15** (Table 8). The crosspeaks at δ 8.00/160.8 (H2′,6′/C4′), δ 8.00/163.2 (H2′,6′/C2), δ 7.99/161.7 (H2‴,6‴/C4‴), δ 7.99/164.7 (H2‴,6‴/C2″), δ 7.06/160.8 (H3′,5′/C4′), δ 7.06/124.5 (H3′,5′/C1′), δ 6.96/161.7 (H3‴,5‴/C4‴), δ 6.96/121.1 (H3‴,5‴/C1‴), δ 7.19/153.6 (H8″/C9″), δ 7.19/156.0 (H8″/C7″), δ 7.19/182.3 (H8″/C4′’), δ 7.19/125.9 (H8″/C6″), δ 7.19/105.8 (H8″/C10″), δ 6.49/157.4 (H8/C9), δ 6.49/164.4 (H8/C7), δ 6.49/103.8 (H8/C10), δ 6.49/99.0 (H8/C6), δ 6.20/164.4 (H6/C7), δ 6.20/161.6 (H6/C5), δ 6.20/103.8 (H6/C10), δ 6.20/94.1 (H6/C8), δ 6.86/163.2 (H3/C2), δ 6.86/181.9 (H3/C2), δ 6.93/164.7 (H3″/C2″), δ 6.93/182.3 (H3″/C2″), δ 12.88/99.0 (5-OH/C6), δ 12.88/161.6 (5-OH/C5), δ 12.88/103.8 (5-OH/C10), δ 10.85/164.4 (7-OH/C7), δ 10.85/99.0 (7-OH/C6), δ 10.85/94.1 (7-OH/C8), δ 13.13/105.8 (5″-OH/C10″), δ 13.13/125.9 (5″-OH/C6″), δ 13.13/152.6 (5″-OH/C5″), δ 10.44/161.7 (4‴-OH/C4‴), and δ 10.44/116.1 (4‴-OH/C4‴) were particularly important in assignments of the individual ^13^C resonances of hinokiflavone aglycone. A crosspeak at δ 13.13/7.06 (5″-OH/H3′,5′) observed in the 2D ^1^H-^1^H ROESY spectrum of **15** confirmed that the flavone monomers were attached to each other between C4′ and C6″ by an ether bond (Figure 3 and Figure S7). The sugar unit of **15** was identified as glucose by the seven ^1^H resonances observed in the 1D ^1^H spectrum, which were assigned by the 1D ^1^H selective TOCSY spectrum (Figure S2), the 2D ^1^H-^1^H COSY spectrum (Figure S6), and the 2D ^1^H-^13^C H2BC spectrum (Figure S5) of **15**, in addition to the six ^13^C signals belonging to the glucosyl unit, which were then assigned by the 2D ^1^H-^13^C HSQC spectrum. A crosspeak at δ 5.17/164.4 (H1‴/C7″) (Figure 3) confirmed that the glucosyl substituent was attached to hinokiflavone aglycone at the 7″-position. Thus, **15** was identified to be the novel biflavonoid hinokiflavone 7″-*O*-*β*-glucopyranoside (Figure 2). A molecular ion [MH^+^] at *m*/*z* 701.15109 corresponding to C_36_H_29_O_15_ (calculated: 701.15065; δ = 0.63 ppm) observed in the high-resolution mass spectrum of **15** (Figure S8) confirmed this identification.

The cytotoxic activities of the isolated compounds against MOLM-13 leukemia cells were determined (Table 9). The majority of the compounds exhibited low-to-moderate cytotoxicity (Table 9). Compounds **8** and **14** were the most potent compounds, which exhibited EC_50_ values at 45–90 µM and 22–44 µM, respectively, after 72 h of incubation (Table 9).

## 3. Discussion

For the first time, glycosides of natural products have been identified in *C. japonica*. Compounds **3**, **4**, **5**, **6**, **7**, **8**, **9**, **12**, **13m**, **14**, and **16** are identified in *C. japonica* for the first time. The rare natural product Mururin A (**16**) has previously only been identified in *Brosimum acutifolium* Huber [49], while catechin-(7,8)-7″-(3,4-dihydroxyphenyl)-dihydro-8″(3H)-pyranone (**14**) has previously been reported from *Phyllocladus trichomanoides* D.Don. [50]. The fact that the majority of the natural products identified in this study have been found in *C. japonica* for the first time may be correlated with the influence of factors such as unusual day lengths during the growth season in Norway, compared with other localities, on the selective production of natural products of *C. japonica*. A similar phenomenon was observed by Nguyen et al. (2014) on the production of natural products of *Metasequoia glyptostroboides* Hu & W.C. Cheng [51]. The isolated compounds exhibited moderate-to-low cytotoxic activity against MOLM-13 leukemia cells, but some compounds, like **14** and **8**, should be investigated as possible candidates for modifications and SAR studies to generate more potent analogues. Most new anti-cancer compounds have activity in the low μM or even nM range, and it would be desirable to lower the EC_50_ values of compound **14** and **8**. If the efficacy can be improved and the selectivity towards cancer cell lines over normal cell lines maintained, these compounds could be further tested for anti-cancer activity towards a panel of cell lines, and eventually in in vivo models for the most relevant cancer. Also, more studies on the cellular mechanism of action can gain insight into targets and possible medical applications. Hinokiflavone 7″-glucoside (compound **15**), which is the first novel flavonoid identified in *C. japonica*, is a biflavonoid glucoside based on the biflavonoid aglycone 4′,6”-*O*-Biapigenin. NMR data of the core structure were in accordance with the literature data for hinokiflavone [52,53]. In the previous literature, hinokiflavone aglycone has been reported to exhibit significant cytotoxic activity against several cancer cell lines [54]. In vivo metabolic activation of the cytotoxic potential of Hinokiflavone 7″-glucoside through de-glycosylation of the compound in the liver to afford hinokiflavone aglycone as a product may be expectable. The cytotoxicity of the common reference compound quercetin (compound **1** in Table 9) was in accordance with what has been reported for this compound previously [55].

## 4. Materials and Methods

### 4.1. Plant Material

*Cryptomeria japonica* was harvested from the Arboretum at Milde near Bergen on 29 September 2020. The trees are grown from seeds collected on mount Tateyama (1400 moh) on the island Honshu in Japan during an expedition in 1976 [56]. The plant material was placed in a freezer maintained at −25 °C for 48 h. Subsequently, the plant was divided, and both needles, stems, and seeds were distributed into six containers of various sizes. The total weight of the utilized plant material before extraction was 4143 g.

### 4.2. Extraction and Purification by Partition with Organic Solvents

The plant material of *Cryptomeria japonica* (4.14 kg) was extracted twice with a total of 50 L HPLC-grade methanol (Sigma-Aldrich, St. Louis, MO, USA), first for 90 h and then for 48 h at room temperature. The extract was then percolated using a glass funnel with glass wool filter and then concentrated with rotary evaporators at a reduced pressure with the water bath temperature at 28 °C. The resulting concentrated aqueous extract (1.75 L) was then purified to partition twice with petroleum ether (Petroleum ether–ACS reagent, Sigma-Aldrich, Saint Louis, MO, USA) in a separation funnel using a total volume of 4.25 L petroleum ether. The remaining water phase after both extractions with petroleum ether amounting to approximately 1350 mL was then concentrated to 1250 mL on a rotavapor to remove traces of petroleum ether.

The resulting water phase (1.25 L) was further purified to partition twice with equal volumes of ethyl acetate (Ethyl Acetate—ACS reagent ≥ 99.5%, Sigma-Aldrich, Saint Louis, MO, USA) in a separation funnel. The residual water phase and the ethyl acetate phase were then concentrated on rotavapor to volumes of 188 mL and 220 mL, respectively.

### 4.3. Amberlite XAD-7 Chromatography

Both the water phase (188 mL) and the ethyl acetate phase (220 mL) were individually further purified by Amberlite XAD-7 column chromatography (column dimensions 50 × 1000 mm, containing 500 g Amberlite^®^ XAD-7, 20–60 mesh, Sigma-Aldrich, Saint Louis, MO, USA) to remove bulk components such as mono- and oligosaccharides, polysaccharides, and aliphatic amino acids from the extract when distilled water was used as the mobile phase [57]. Under these solvent conditions, the XAD-7 column material absorbs fewer polar and aromatic compounds. The latter compounds were then eluted from the XAD-7 column when the mobile phase was changed to pure methanol (HPLC grade). The mobile phase composition applied during separation of the water phase consisted of 5.0 L distilled water, followed by 3.0 L methanol, while the mobile phase composition applied during separation of the ethyl acetate phase consisted of 5.0 L distilled water followed by 5.0 L methanol. XAD-7 chromatographic separation of the water phase gave a total of 9 fractions where the volumes of fractions 1–5 were 1L, and the volumes of fractions 6–9 were 0.5 L. XAD-7 chromatographic separation of the ethyl acetate phase gave a total of 10 phases where the volumes of fractions 1–5 were 1L, and the volumes of fractions 6–10 were 0.5 L. All fractions were analyzed by analytical HPLC.

### 4.4. Sephadex LH-20 Gel Filtration Chromatography

Based on results from analytical HPLC, different fractions obtained from the XAD-7 purification fractions were selected for further separation with gel filtration chromatography with Sephadex LH-20 columns. Multiple fractions were consolidated based on the apparent presence of the same compounds, aiming to enhance the concentration and thereby increase the mass available for isolation. The consolidation included water phase fractions 6 and 7 (WP6-7) which constituted 1150 mL, ethyl acetate fractions 5 and 6 (EA5-6) which constituted 925 mL, and 8, 9, and 10 (EA8-10) which constituted 2450 mL. Additionally, ethyl acetate fraction 7 (EA7), which constituted 400 mL, was chosen for further purification. WP6-7 and EA5-6 were concentrated to, respectively, 47 mL and 53 mL before being applied to the larger Sephadex LH-20 column (column dimensions 50 × 1000 mm, containing 500 g of Sephadex^®^ LH-20, Sigma-Aldrich, Saint Louis, MO, USA), while EA7 and EA8-10 were concentrated to, respectively, 10 and 22 mL before being applied to the smaller Sephadex LH-20 column (column dimensions 35 × 500 mm, containing 500 g of Sephadex^®^ LH-20, Sigma-Aldrich, Saint Louis, MO, USA). The fractions were eluted from the column using a mobile phase consisting of one part ultra-purified water and one part methanol, along with 0.1% trifluoroacetic acid (TFA). The different mobile phases were water–methanol–TFA 80:20:0.1 *v*/*v*/*v* (A), water–methanol–TFA 50:50:0.1 *v*/*v*/*v* (B), water–methanol–TFA 30:70:0.1 *v*/*v*/*v* (C), and finally methanol–TFA 100:0.1 *v*/*v* (D). During the separation of WP6-7 and EA5-6, acetone (HPLC-quality, Sigma-Aldrich, St. Louis, MO, USA) was also employed as a mobile phase to potentially capture highly retarded compounds. For separation of WP6-7, 2000 mL of mobile phase A, 3000 mL of mobile phase B, 6000 mL of mobile phase C, 6000 mL of mobile phase D, and 2000 mL of acetone were used, resulting in 329 fractions collected. For separation of EA5-6, 3000 mL of mobile phase A, 4000 mL of mobile phase B, 5500 mL of mobile phase C, 11,800 mL of mobile phase D, and 1200 mL of acetone were used, resulting in 333 fractions collected. For separation of EA7, 400 mL of mobile phase A, 1000 mL of mobile phase B, 1350 mL of mobile phase C, and 400 mL of mobile phase D were used, with no acetone applied, resulting in 68 fractions collected. For separation of EA8-10, 340 mL of mobile phase A, 200 mL of mobile phase B, 510 mL of mobile phase C, and 1980 mL of mobile phase D were used, with no acetone applied, resulting in 40 fractions collected. Fractions were collected in vials with volumes ranging from 10 to 20 mL. Pure taxifolin 7-*O*-*β*-glucopyranoside (compound **5**) was isolated in fraction 66–68 of VF6+7, a mix of chlorogenic acid (compound **13**) and methyl chlorogenate (compound **13m**) was isolated in fraction 75–82 of WP6-7, while pure taxifolin 3-*O*-*β*-glucopyranoside (compound **4**) was isolated from both WP6-7 93-94 and EA5-6 fractions 15–18.

### 4.5. Spectroscopy

The mass spectra of compounds **14** and **15** mass were recorded using an HRMS JEOL AccuTOF™ JMS T100LC instrument fitted with an electrospray ion source operated in positive mode at a resolving power of approximately 6000 FWHM. The spectrum was recorded over the mass range of 50–2000 *m*/*z*. The samples were analyzed as solutions in methanol and introduced to the ESI spray chamber by weakly acidified (0.01% HCOOH) acetonitrile (Acetonitrile—for HPLC, gradient grade, ≥99.9%, Sigma-Aldrich, Saint Louis, MO, USA) used as a spray reagent. Ultra-performance liquid chromatography coupled with high-resolution mass spectrometry (UPLC-HRMS) was also used for the exact mass determination of compound **16**. An iClass UPLC (Waters) equipped with a C_18_ BEH column (1.7 um, 2.1 × 50 mm, Waters) was used for introducing the samples to the mass spectrometer. A gradient of (A) 0.2% formic acid and (B) acetonitrile was used as follows (% B in A): 1 (isocratic for 0.5 min), from 1 to 90 (2 min). The mass spectrometer (timsTOF, Bruker, Preston, VIC, Australia) was used in ESI+-mode with an ionization at 2 kV and with full scan 100–2000 Da with resolution R = 50,000 (FWHM) at 1000 Da. Exactness at RMS < 1 ppm.

UV-Vis absorption spectra were recorded online during the analytical HPLC analysis over the 210–600 nm wavelength range in steps of 2 nm.

NMR samples were prepared by individually dissolving isolated compounds in deuterated dimethylsulfoxide (DMSO-D_6_; 99.96 atom% D, Sigma-Aldrich, Saint Louis, MO, USA). The 1D ^1^H, 1D ^1^H selective TOCSY, 1D ^13^CAPT and the 2D ^1^H-^13^C HMBC, the 2D ^1^H-^13^C HSQC, ^1^H-^13^C HSQC-TOCSY, the 2D ^1^H-^13^C H2BC, and the 2D ^1^H-^1^H COSY and 2D ^1^H-^1^H ROESY NMR experiments were obtained on a Bruker BioSpin AVANCE III HD 850 MHz (Fällanden, Switzerland) equipped with a ^1^H, ^13^C, and ^15^N triple resonance cryogenic probe at 298 K.

### 4.6. Cytotoxicity

Pure compounds were dissolved in DMSO (Sigma-Aldrich, Saint Louis, MO, USA) to create stock solutions with a final concentration of 20 mM.

The AML cell line MOLM13 (DSMZ no.: ACC554, [58] was maintained in RPMI-1640 medium supplemented with 10% FBS and 8mM L-glutamine (Sigma Life Science, Dorset, UK) and 1 IU/mL penicillin and 1 mg/mL streptomycin (Cambrex, Villers-le-Bouillet, Belgium) were added. The cells were kept in suspension cultures with a density between 150,000 and 700,000 cells/mL.

The cells were incubated in a humidified environment (37 °C, 5% CO_2_). For the cytotoxicity experiments, the MOLM13 cells were seeded in 96-well tissue culture plates at 20,000 cells/well with 0.1 mL on the day of the experiment.

Compounds dissolved in DMSO were added to the cells, and the plates were incubated for 72 h before adding the tetrazolium salt WST-1 according to the manufacturer’s instructions (Roche Diagnostics GmbH Mannheim, Mannheim, Germany). The plates were further incubated for two hours before the signal was recorded at 450 nm with reference at 620 nm. For black subtraction, medium and plant compounds were added to WST-1. This procedure was conducted after 24 and 72 h.

After recording WST-1, the cells were next fixed with 2% buffered formaldehyde (pH 7.4) with 0.01 mg/mL of the DNA-specific fluorescent dye, Hoechst 33342. As previously described, the presence of dead (apoptotic or necrotic) cells was verified by differential interference contrast and fluorescence microscopy [59,60].

EC_50_ values were determined by a four-parameter regression analysis as described by Viktorsson et al. [61], using SigmaPlot ver. 14.0 (Systat Software Inc., San Jose, CA, USA).

The cells were routinely tested for the presence of mycoplasma every six to eight weeks, using MycoAlert™ (Lonza Rockland, Inc., Rockland, ME, USA). No mycoplasma infection was detected during this study.

## 5. Conclusions

*Cryptomeria japonica* continues to be a promising source of novel natural products. The majority of the compounds reported in this paper are characterized from this species for the first time, including the first identification of glycosides from *C. japonica*. The fact that a novel biflavonoid glucoside for the first time has been isolated from this species, in addition to several natural products belonging to compound classes which have not previously been identified from *C. japonica*, encourages further studies to reveal if these compounds may play a potential role with respect to the positive health effects associated with the applications of *C. japonica* in traditional medicine.

## Figures and Tables

**Table 1:** Monoterpenoids and other volatile compounds previously identified from *Cryptomeria japonica*.

Compound	Method	References	Compound	Method	References
(–)-Bornyl acetate	NMR, GC-MS	[14,15,16,17,18]	Myrtenal	GC-MS	[14,15]
(cis)-Verbenone	GC-MS	[14,15,19]	n-Hexanol	GC-MS	[14,15]
1-Octen-3-ol	GC-MS	[14,15]	Nopol	GC-MS	[16]
1-Octene-3-yl acetate	GC-MS	[14,15]	p-Cymene	NMR, GC-MS	[14,15,16,17,18]
2-p-Menthen-1-ol	NMR, GC-MS	[18]	Pinocarvone	GC-MS	[19]
artemiseole	GC-MS	[16]	Pinol	GC-MS	[19]
Azulene	GC-MS	[16]	Piperitone	GC-MS	[19]
Borneol	GC-MS	[20]	Pulegone	GC-MS	[14,15]
bornylene	GC-MS	[16]	Sabinene	NMR, GC-MS	[14,15,16,17,18,19,20,21]
Camphene	NMR, GC-MS	[14,15,17,18,19,20]	Terpinen-4-ol	NMR, GC-MS	[14,15,16,18,22]
Camphor	GC-MS	[19]	Thymol	GC-MS	[14,15,19]
Carvacrol	GC-MS	[19]	Trans-geraniol	GC-MS	[19]
Carveol	GC-MS	[19]	trans-Pinocarveol	GC-MS	[14,15,19]
Carvone	GC-MS	[14,15,19]	trans-Piperitol	GC-MS	[14,15]
Cineol	GC-MS	[19]	trans-p-menth-2-enol	GC-MS	[19]
cis-3-Hexen-1-ol	GC-MS	[14,15,19]	trans-2-p-Menthen-1-ol	NMR, GC-MS	[23]
cis-Sabinene hydrate	GC-MS	[14,15]	trans-Sabinene hydrate	GC-MS	[14,15]
Cuminol	GC-MS	[19]	Tricyclene	NMR, GC-MS	[14,15,18,19]
dehydro-p-cymene	GC-MS	[16]	Verbenone	GC-MS	[20]
Elemol acetat	GC-MS	[17]	α-Fencol	GC-MS	[19,20]
eucarvone	GC-MS	[16]	α-Phellandrene	GC-MS	[14,15,19]
Eugenol	GC-MS	[19]	α-Pinene	NMR, GC-MS	[14,15,16,17,18,19,20,21]
Fenchone	GC-MS	[19]	α-Terpinene	NMR, GC-MS	[14,16,17,18,19,20]
Geranyl acetat	GC-MS	[19]	α-Terpineol	NMR, GC-MS	[16,18,19,20,21,22]
Ipsdienol	GC-MS	[19]	α-Terpinolene	GC-MS	[14,15,16,17,19,20]
iso-Bornyl acetate	GC-MS	[14,15]	α-Terpinyl acetate	GC-MS	[20]
Isocineole	GC-MS	[19]	α-Thujene	NMR, GC-MS	[14,15,17,18,19]
isopulegol acetate	GC-MS	[16]	β-Myrcene	NMR, GC-MS	[17,18,19,21]
L-Borneol	GC-MS	[14,15]	β-Phellandrene	GC-MS	[14,15]
Limonene	NMR, GC-MS	[14,15,16,17,18,19,20,21]	β-Pinene	NMR, GC-MS	[14,15,16,17,18,19,20,21]
Linalool	GC-MS	[14,15,19]	β-Terpinyl acetate	GC-MS	[14,15]
Linalyl acetat	GC-MS	[19]	β-Thujene	GC-MS	[20]
m-cymene	GC-MS	[42]	β-Thujone	GC-MS	[14,15,17,18,19]
Methyl carvacrol	GC-MS	[45]	γ-Terpinene	NMR, GC-MS	[14,15,17,18,19,20,21]
Methyleugenol	GC-MS	[45]	δ-3-Carene	NMR, GC-MS	[14,15,16,17,18,19,21]

**Table 2:** Sesquiterpenoids previously isolated from *Cryptomeria japonica*.

Compound	Method	References	Compound	Method	References
(1S,6R)-2,7(14),10-bisabolatrien-1-ol-4-one	NMR, EI-MS	[12]	Germacrene D	NMR, GC-MS	[14,15,16,18,19]
(−)-cubenene	GC-MS	[16,19]	Gleenol	GC-MS	[10]
(+)-cyclosativene	GC-MS	[16]	Globulol	GC-MS	[14,15]
(15)-eudesmene-1 β,11-diol	NMR, EI-MS	[24]	Guaiol	GC-MS	[14,15]
(1S,6R)-2,7(14),10-bisabolatrien-1-ol-4-one	NMR, EI-MS	[7]	Hedycariol	NMR, GC-MS	[18]
(1S,6R)-2,7(14),10-bisabolatrien-1-ol-4-one	GC-MS	[25]	Humulene	GC-MS	[16]
(4S)-2,6,10-bisaboratrien-4-ol-1-one	NMR, EI-MS	[26]	isoledene	GC-MS	[16]
1(10)-Cadinen-4α-ol	GC-MS	[20]	Juniper camphor	GC-MS	[19]
1(10)-Cadinen-4β-ol	GC-MS	[20]	Kongol	GC-MS	[10]
1,1,3a-trimethyl-7-methylene-decahydrocyclopropa[a]-naphthalene	GC-MS	[16]	Longicyclene	GC-MS	[20]
1,8-epoxy-1(6),2,4,7,10-bisaborapentaen-4-ol	NMR, EI-MS	[26]	Longicyclenylalcohol	GC-MS	[20]
1,10-di-epi-Cubenol	GC-MS	[27]	Longifolene	GC-MS	[20]
10(15)-Cadinen-4-ol	GC-MS	[15,20]	Longiisohomocamphenilone	GC-MS	[20]
10-epi-γ-Eudesmol	GC-MS	[17]	Longiverbenone	GC-MS	[16,20]
11-acetoxyeudesman-4α-ol	NMR, EI-MS	[24]	Longi-αnojigikualcohol	GC-MS	[20]
11-hydroxy-4,5-secoeudesmane-4,5-dione	NMR, EI-MS	[24]	Longi-β-camphenilanaldehyde	GC-MS	[20]
1-methoxy-4-cadinene	NMR, EI-MS	[26]	Manoyl oxide	GC-MS	[14,15]
1β-acetoxy-3-eudesmen-11-ol	NMR, EI-MS	[24]	Muurora-3,5-diene	GC-MS	[27]
1β-acetoxy-4(15)-eudesmen-11-ol	NMR, EI-MS	[24]	Nerolidol	NMR, GC-MS	[18,19]
1β-acetoxy-4-eudesmen-11-ol	NMR, EI-MS	[24]	Oplodiol	NMR, EI-MS	[24]
2-methylene-5-(1-methylvinyl)-8-methylbicyclo[5.3.0]decane	GC-MS	[16]	Oplopanone	NMR, EI-MS	[24]
3-eudesmene-1β,11-diol	NMR, EI-MS	[24]	Oplopanonyl acetate	GC-MS	[19]
4-epi-Cubebol	GC-MS	[27]	p-cymen-8-ol	GC-MS	[19]
4-epicryptomeridiol	NMR, EI-MS	[24]	Rimuene	GC-MS	[14,15]
4(15)-eudesmene-1β,6α-diol	NMR, EI-MS	[24]	Spathulenol	GC-MS	[16]
4,4-dimethyl-3-(3-methylbut-3-enylidene)-2-methylene-bicyclo[4.1.0]heptane	GC-MS	[16]	Thujopsene	GC-MS	[14,15,19]
4,5,9,10-dehydroisolongifolene	GC-MS	[16]	Torreyol	GC-MS	[10,14,15]
4-eudesmene-1β,11-diol	NMR, EI-MS	[24]	Trans-γ-bisabolene	GC-MS	[19]
6a,7a-dihydroxyabieta-8,12-dien-11,14-dione	NMR, EI-MS	[28]	trans-β-Farnesene	NMR, GC-MS	[14,15,18,19]
6a,7b-dihydroxyabieta-8,12-diene-11,14-dione	NMR, EI-MS	[28]	valencene	GC-MS	[16]
6-eudesmene-1 β,4 β-diol	NMR, EI-MS	[24]	Viridiflorol	GC-MS	[14,15]
7a-butoxyabieta-8,12-diene-11,14-dione	NMR, EI-MS, IR	[28]	α-caryophyllene	GC-MS	[16,19]
7-epi-4-eudesmene-1β,11-diol	NMR, EI-MS	[24]	α-longipinene	GC-MS	[19,20]
7-epi-γ-eudesmol	NMR, EI-MS	[24]	α-acoradiene	GC-MS	[19]
Allo-Hedycariol	NMR, GC-MS	[18]	α-bisabolol	GC-MS	[19]
ar-Curcumene	GC-MS	[14,15]	α-Cadinene/α-amorphene	GC-MS	[14,15,16]
Aromadendrene	GC-MS	[16]	α-Cadinol	NMR, GC-MS, EI-MS	[14,15,16,17,18,20,24]
Bicyclosesquiphellandrene	GC-MS	[19]	α-Calacorene	NMR, GC-MS	[18,19]
Bulnesol	GC-MS	[17]	α-cedrene	GC-MS	[19]
cadala-1(10),3,8-triene	GC-MS	[16]	α-cedrol	NMR, GC-MS, EI-MS	[19,24]
Cadalene	GC-MS	[16,19]	α-Copaene	NMR, GC-MS	[15,16,18,20]
Cadina-1(6),4-diene	GC-MS	[27]	α-Cubebene	NMR, GC-MS	[10,15,16,18]
cadina-1,3,5-triene	GC-MS	[16]	α-Elemene	GC-MS	[20]
Cadina-1,4-diene	NMR, GC-MS	[18]	α-Eudesmol	NMR, GC-MS, EI-MS	[10,14,15,16,17,18,19,20,21,22,24]
cadina-3,9-diene	GC-MS	[16]	α-guaiene	GC-MS	[16,20]
Calacorene	GC-MS	[20]	α-gurjunene	GC-MS	[16]
Calamenene	GC-MS	[45,46]	α-Humulene	GC-MS	[20]
Caryophyllene oxide	NMR, GC-MS	[16,18,19]	α-Humulene oxide	NMR, GC-MS	[14,15,18]
Cedrol	NMR, GC-MS, IR	[20]	α-Muurolene	NMR, GC-MS	[10,16,17,18,19,20]
cis-β-Farnesol	GC-MS	[14,15,19]	α-selinene	GC-MS	[19]
cis-Caryophyllene	GC-MS	[20]	α-Ylangene	GC-MS	[14,15,16]
cis-Muurola-4(15),5-diene	GC-MS	[27]	α-Zingiberene	GC-MS	[14,15]
cis-β-farnesene	GC-MS	[19]	β-Bisabolene	GC-MS	[27]
Cryptomeridiol	NMR, EI-MS	[24]	β-cedrene	GC-MS	[19]
Cryptomerione	GC-MS	[10]	β-funebrene	GC-MS	[19]
Cubebol	NMR, GC-MS	[18,29]	β-cadinene	GC-MS	[19]
Cubenol	NMR, GC-MS	[10,18,29]	β-Caryophyllene	NMR, GC-MS	[14,15,16,18,19,20]
Cuparene	GC-MS	[19]	β-Cubebene	NMR, GC-MS	[16,18,20]
diepi-α-Cedren	GC-MS	[14,15]	β-Elemene	NMR, GC-MS	[14,15,17,19]
Dihydroeudesmol	GC-MS	[27]	β-Eudesmol	NMR, GC-MS, EI-MS	[10,14,15,17,18,20,21,24]
Dihydrocaryophyllen-5-one	GC-MS	[20]	β-Guaiene	GC-MS	[20]
elem-1-en-4,11-diol	NMR, EI-MS	[24]	β-himachalene	GC-MS	[19]
Elemol	NMR, GC-MS, EI-MS	[10,14,15,17,18,19,20,21,22,24]	β-Selinene	GC-MS	[19]
Elemyl acetate	GC-MS	[14,15]	β-Sesquiphellandren	GC-MS	[14,15]
epi-bicyclosesquiphellandrene	NMR, GC-MS	[16,18]	γ-Cadinene	NMR, GC-MS	[16,17,18,19,20]
epi-Cubebol	NMR, GC-MS	[18]	γ-Elemene	GC-MS	[14,15,16]
Epi-cubenol	GC-MS	[10]	γ-Eudesmol	NMR, GC-MS, EI-MS, IR	[10,17,18,19,20,21,22]
Epijuvabione	NMR, EI-MS	[24]	γ-Muurolene	NMR, GC-MS	[14,15,16,17,18,19,20]
Epitodomatuic acid	NMR, EI-MS	[24]	δ-guaiene	GC-MS	[19]
epi-Zonarene	NMR, GC-MS	[18]	δ-Cadiene	GC-MS	[14,15,17,19]
eudesma-3,7(11)-diene	GC-MS	[16]	δ-Cadinene	NMR, GC-MS	[10,18,22]
eudesmane-5 α,11-diol	NMR, EI-MS	[24]	δ-cadinol	NMR, GC-MS	[29]
ferrugicadinol	NMR, EI-MS, IR	[30]	δ-Elemene	GC-MS	[19]
ferrugicadinol A	NMR, EI-MS, IR	[30]	δ-Selinene	GC-MS	[14,15]
ferrugicryptomeridiol	NMR, EI-MS, IR	[30]	τ-Cadinol	NMR, GC-MS, EI-MS	[10,14,17,18]
Germacrene B	GC-MS	[14,15,19]	τ-metropol	NMR, GC-MS	[17,18,20]

**Table 3:** Diterpenoids previously isolated from *Cryptomeria japonica*.

Compound	Method	References	Compound	Method	References
(+)-16-acetylkaurane-16,17-diol	NMR, EI-MS	[31]	Cryptotrione	NMR, EI-MS, IR	[5]
(+)-Phyllocladen	NMR, GC-MS	[18]	Cupresol	NMR, EI-MS, IR	[32,33]
(5R,10S)-12-methoxyabieta-6,8,11,13-tetraene	NMR	[31]	Cupressene	NMR, GC-MS	[16,18]
11-hydroxysugiol	NMR, EI-MS, IR	[31]	Dehydroferruginol	NMR, GC-MS	[18]
12-hydroxy-11-methoxyabieta-8,11,13-trien-7-one	NMR, EI-MS, IR	[32]	ent-17-norkauran-16-one	NMR	[31]
12-hydroxy-6,7-secoabieta-8,11,13-triene-6,7-dial	NMR, EI-MS, IR	[31]	ent-kaur-15-en-17-al	NMR	[31]
13-epicupressic acid methyl ester	NMR, EI-MS, IR	[32]	ent-kaur-15-en-17-ol	NMR	[31]
13-isopimaradiene	GC-MS	[19]	ent-kaur-16-ene	GC-MS	[16]
13-oxo-14,15-dinorlabd-8(17)-en-19-oic acid methyl ester	NMR, EI-MS, IR	[32]	Ferruginol	NMR, GC-MS	[8,16,18,29,31,33,34]
15-Kaurene	GC-MS	[17]	hinokiol	NMR, EI-MS, IR	[31]
16-Kaurene	GC-MS	[17]	Iguestol	NMR	[8]
18-nor-isopimar-4(19),7,15-triene	GC-MS	[16]	Isopimaradiene	NMR, GC-MS	[18]
19-acetoxyferruginol	NMR, EI-MS, IR	[32]	isopimaric acid	NMR, EI-MS, IR	[8,32]
1-epicubenol	NMR, GC-MS	[29]	isopimarinol	NMR, EI-MS, IR	[32]
4-epicubenol	NMR, GC-MS	[29]	Isopimarol	NMR	[8]
5,6-dehydrosugiol methyl ether	NMR, EI-MS, IR	[32]	Isopressic acid	NMR	[33]
5-epixanthoperol	NMR, EI-MS, IR	[31,33]	Junicedric acid	NMR, EI-MS, IR	[32]
6,12-dihydroxy-11-methoxyabieta-5,8,11,13-tetraen-7-one	NMR, EI-MS, IR	[32]	Kaur-16-ene	GC-MS	[19]
6,12-Dihydroxy-5,8,11,13-abietatetraen-7-one	NMR	[33]	Kauran-16-ol	NMR, GC-MS	[16,18]
6,12-dihydroxyabieta-5,8,11,13-tetraen-7-one	NMR, EI-MS	[31]	Kaurene	GC-MS	[19,21,22]
6,7-dehydroferruginol	NMR, EI-MS, IR	[31]	Nezukol	NMR, GC-MS, EI-MS, IR	[17,22,32]
6,7-dehydroferruginol methyl ether	NMR, EI-MS, IR	[31]	Pimara-8(14),15-diene	NMR, GC-MS	[18]
6-hydroxy-5,6-dehydrosugiol	NMR	[8]	Pimara-8,15-diene	NMR, GC-MS	[18]
6α,11-dihydroxy-12-methoxyabieta-8,11,13-trien-7-one	NMR, EI-MS, IR	[32]	Phyllocradanol	GC-MS	[29]
6α-hydroxydemethylcryptojaponol	NMR, EI-MS, IR	[32]	Phytane	GC-MS	[19]
6α-hydroxysugiol	NMR, EI-MS, IR	[31]	Phytol	NMR, EI-MS, IR	[32]
6β-hydroxyferruginol	NMR, EI-MS	[31]	Pimarinal	GC-MS	[16]
7-dehydroabietanone	NMR, EI-MS, IR	[31]	Sandaracopimaradiene	NMR, GC-MS	[16,18]
7α,8α-epoxy-6α-hydroxyabetia-9(11),13-dien-12one	NMR, EI-MS	[31]	sandaracopimaric acid	NMR, EI-MS, IR	[32]
8,13-dioxo-14,15,17-trinorlabdan-19-oic acid	NMR, EI-MS, IR	[32]	sandaracopimarinal	NMR, GC-MS	[18,29]
8-β-hydroxyabieta-9(11),13-dien-12-one	NMR	[33]	Sandaracopimarinol	NMR, GC-MS	[8,18,29]
8β-Hydroxysandaracopimarene	NMR, GC-MS	[18]	Sclarene	GC-MS	[16,19]
abieta-8,11,13-triene	GC-MS	[19,34]	Sclareol	GC-MS	[16]
Abietadiene	GC-MS	[27]	secoabietane dialdehyd	NMR, EI-MS, IR	[31]
abietatriene	GC-MS	[16]	Sugiol	NMR, EI-MS, IR	[7,8,31,33]
cis-communic acid	NMR, EI-MS, IR	[32]	sugiol methyl esther	NMR, EI-MS, IR	[32]
Copalol	NMR, EI-MS, IR	[32]	Totarol	NMR, GC-MS, IR	[16,31]
cryptojaponol	NMR, EI-MS, IR	[31,33]	trans-communic acid	NMR, EI-MS, IR	[32]
Cryptoquinone	NMR, EI-MS,	[9]	Verticiol	GC-MS	[16]

**Table 4:** Triterpenoids previously identified in *Cryptomeria japonica*.

Compound	Method	References
10′-oxocryptoquinone	EI-MS	[35]
10′β-hydroxycryptoquinone	NMR, EI-MS, IR	[35]
13-methyl-17-norkaur-15-en	GC-MS	[15]
6α-hydroxychamaecydin	NMR, EI-MS, IR	[35]
6β-hydroxychamaecydin	EI-MS	[35]
Chamaecydin	NMR, EI-MS	[35]

**Table 5:** Flavonoids previously identified in *Cryptomeria japonica*.

Compound	Method	References	Compound	Method	References
2,3-dihydroamentoflavone	NMR, FD-MS, IR	[36]	Epicatechol pentaacetat	NMR, IR	[37]
4′,4‴,7,7″-tetrametylamentoflavone	NMR, IR	[37]	Gallocatechin	2D HPLC	[38]
5‴-hydroxyamentoflavone	NMR, FD-MS	[36]	Luteolin	NMR, FD-MS	[36]
5-hydroxy-3,4′,7-trimetoxyflavone	NMR, IR	[37]	Naringenin	NMR, FD-MS	[36]
5-hydroxy-4′-7-dimetoxyflavone	NMR, IR	[37]	Procyanidin B-1	NMR	[39]
Afzelin	NMR, FD-MS	[36]	Procyanidin B-2	NMR	[39]
Amentoflavone	NMR, FD-MS	[36]	Procyanidin B-3	NMR, GC-MS	[29,40]
Apigenin	NMR, FD-MS	[36]	Procyanidin B-4	NMR	[39]
Catechin	NMR, GC-MS	[29,40,41]	Quercetin	NMR, GC-MS, IR	[37]
Catechol pentaacetat	NMR, IR	[37]	Quercitrin	NMR, FD-MS	[36]
Cosmosiin	NMR, FD-MS	[36]	Taxifolin	NMR, IR	[37]
Epicatechin	NMR, GC-MS	[29]			

**Table 6:** Lignans previously identified in *Cryptomeria japonica*.

Compound	Method	References	Compound	Method	References
Agatharesinol	GC-MS	[42]	(−)-matairesinol	NMR, TLC	[42]
Agatharesinol tetraacetat	NMR, IR	[37]	Matairesinol	NMR, IR	[37]
Cedrusin tetraacetat	NMR, IR	[37]	Notrachelogenin	NMR, IR	[37]
Cedrusinin triacetat	NMR, IR	[37]	(−)-pinoresinol	NMR, TLC	[42]
Cis-dihydrodehydrodiconiferyl alcohol triacetat	NMR, IR	[37]	Secodihydrodehydrodiconiferyl alcohol tetraacetat	NMR, IR	[37]
Dihydrodehydrodiconiferyl alcohol triacetat	NMR, IR	[37]	Secoisolariciresinol tetraacetat	NMR, IR	[37]
Isolariciresinol tetraacetat	NMR, IR	[37]			

**Table 7:** Other compounds previously identified in *Cryptomeria japonica*.

Compound	Method	References
2,5-di-tert-butylphenol	GC-MS	[16]
4-β-D-glucopyranosyloxyferulic acid	NMR	[36]
6-(2-butenyl)-1,5,5-trimethyl-cyclohexene	GC-MS	[16]
Arabinopyranose	GC-MS	[41]
Caladene	NMR, GC-MS	[18]
Chlorogenic acid	GC-MS	[41]
D-Pinitol	GC-MS	[41]
Deoxyoblongifolion	GC-MS	[16]
Ferulic acid	IR	[36]
Fructopyranose	GC-MS	[41]
Glucopyranose	GC-MS	[41]
Isopimaric acid	GC-MS	[41]
Mannopyranose	GC-MS	[41]
Shikimic acid	GC-MS	[41]
Tremetone	NMR, GC-MS	[18]
β-maaiene	GC-MS	[16]
β-methylallylbenzene	GC-MS	[16]
*ρ*-coumaric acid	IR	[36]

**Table 8:** ^1^H and ^13^C NMR chemical shift values (ppm) and coupling constants (Hz) for hinokiflavone 7″-*O*-*β*-glucopyranoside (**15**) in DMSO-D_6_ at 298 K.

	δ ^1^H	δ ^13^C
2		163.2
3	6.86 s	104.1
4		181.9
5		161.6
6	6.20 d 2.1	99.0
7		164.4
8	6.49 d 2.1	94.1
9		157.4
10		103.8
1′		124.5
2′/6′	8.00 ‘d’ 9.0	128.5
3′/5′	7.06 ‘d’ 9.0	115.8
4′		160.8
5-OH	12.88 s	
7-OH	10.85 s	
2″		164.7
3″	6.93 s	102.9
4′’		182.3
5″		152.6
6″		125.9
7″		156.0
8″	7.19 s	94.8
9″		153.6
10″		105.8
1‴		121.1
2‴/6‴	7.99 ‘d’ 8.9	128.8
3‴/5‴	6.96 ‘d’ 8.9	116.1
4‴		161.7
5″-OH	13.13 s	
4‴-OH	10.44 s	
7″-*O*-*β*-glucopyranoside		
1‴	5.17 d 7.7	100.2
2‴	3.11 m	73.1
3‴	3.25 t 9.0	76.8
4′‴	3.11 t 9.0	69.5
5‴	3.45 m	77.5
6A‴	3.70 m	60.6
6B‴	3.45 m	

**Table 9:** EC_50_-values of compounds **1**–**16** for MOLM-13 cell line.

Compound	EC_50_ Values (µM) for MOLM-13 Leukemia Cells After 72 h Incubation
**1**	60–120
**2**	n.d.
**3**	333–665
**4**	325–650
**5**	165–330
**6**	17.5–35
**7**	52–104
**8**	45–90
**9**	n.d.
**10**	143
**11**	n.d.
**12**	n.d.
**13**	411.25–822.5
**14**	22–44
**15**	n.d.
**16**	95–190

## Data Availability

The original contributions presented in this study are included in this article/Supplementary Materials. Further inquiries can be directed to the corresponding author.

## Supplementary Materials

The following supporting information can be downloaded at: https://www.mdpi.com/article/10.3390/ijms252413735/s1.
