# Supplementary material for: Cytotoxic Natural Products from Cryptomeria japonica (Thunb. ex L.) D.Don
Source: Int J Mol Sci. 2024 Dec 23;25(24):13735. doi: 10.3390/ijms252413735 (PMC11677600; doi:10.3390/ijms252413735)
Supplement: Supplementary file 1 [file ijms-25-13735-s001.zip › ijms-3374294-supplementary.pdf]

Cytotoxic natural products from *Cryptomeria japonica* (Thunb. ex L.) D.Don

Supplementary data

Fig. S1. 1D ¹H NMR spectrum of hinokiflavone 7''-*O*-*β*-glucopyranoside

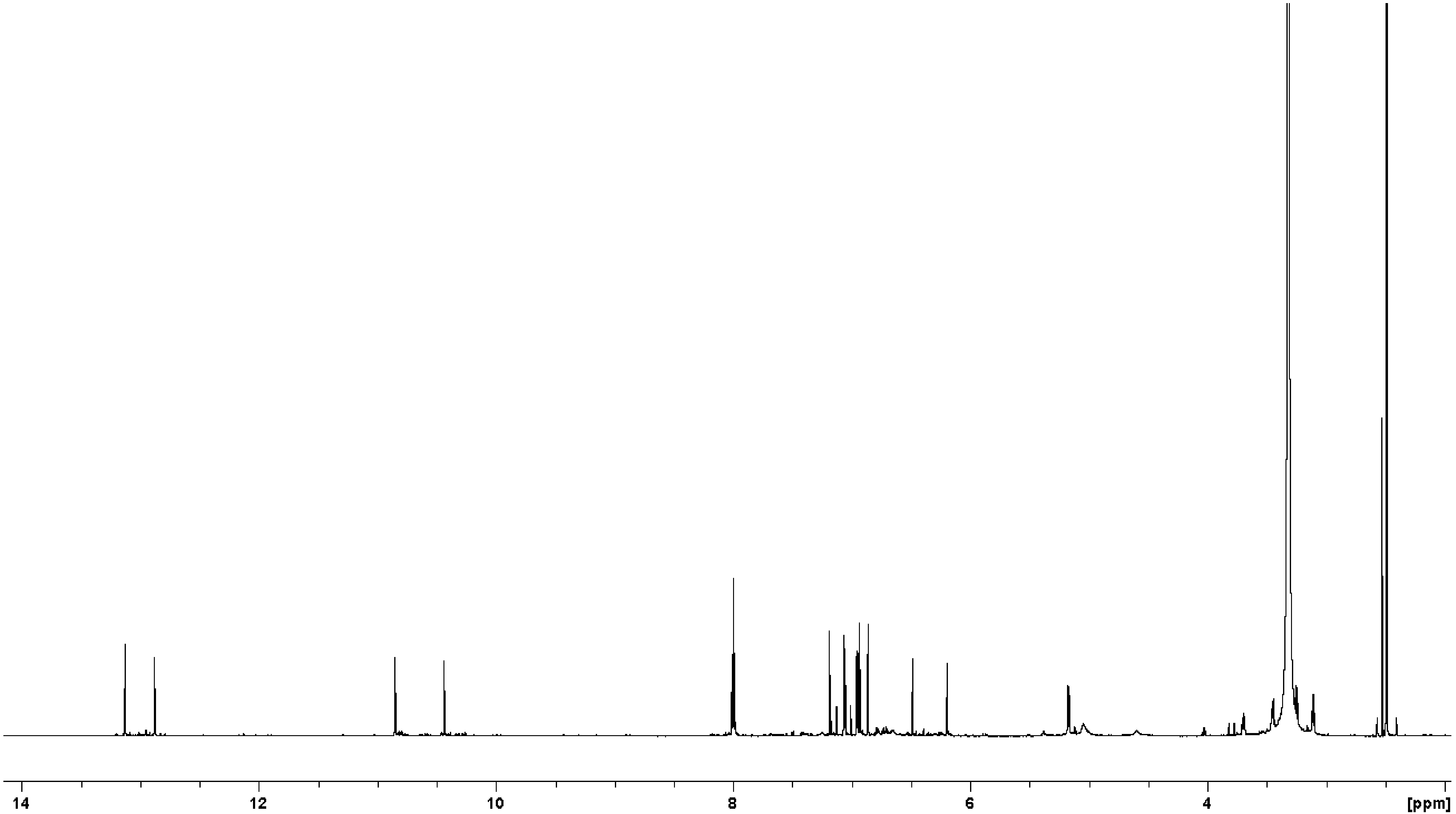

Fig. S2. 1D ^{1}H selective TOCSY NMR spectrum of the 3-glucopyranosyl unit of hinokiflavone 7''-*O*-*β*-glucopyranoside

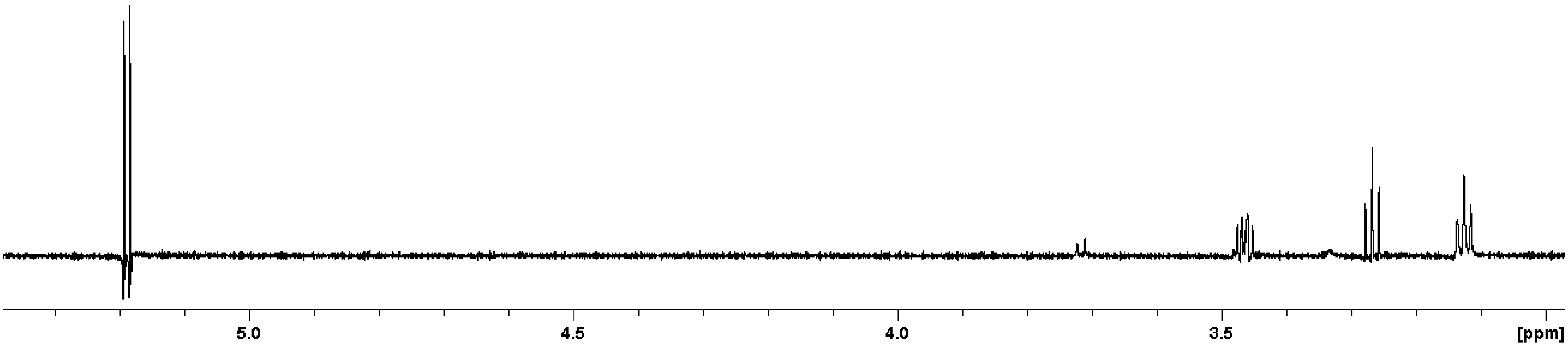

Fig. S3. 2D ^{1}H-^{13}C HMBC NMR spectrum of hinokiflavone 7''-*O*-*β*-glucopyranoside

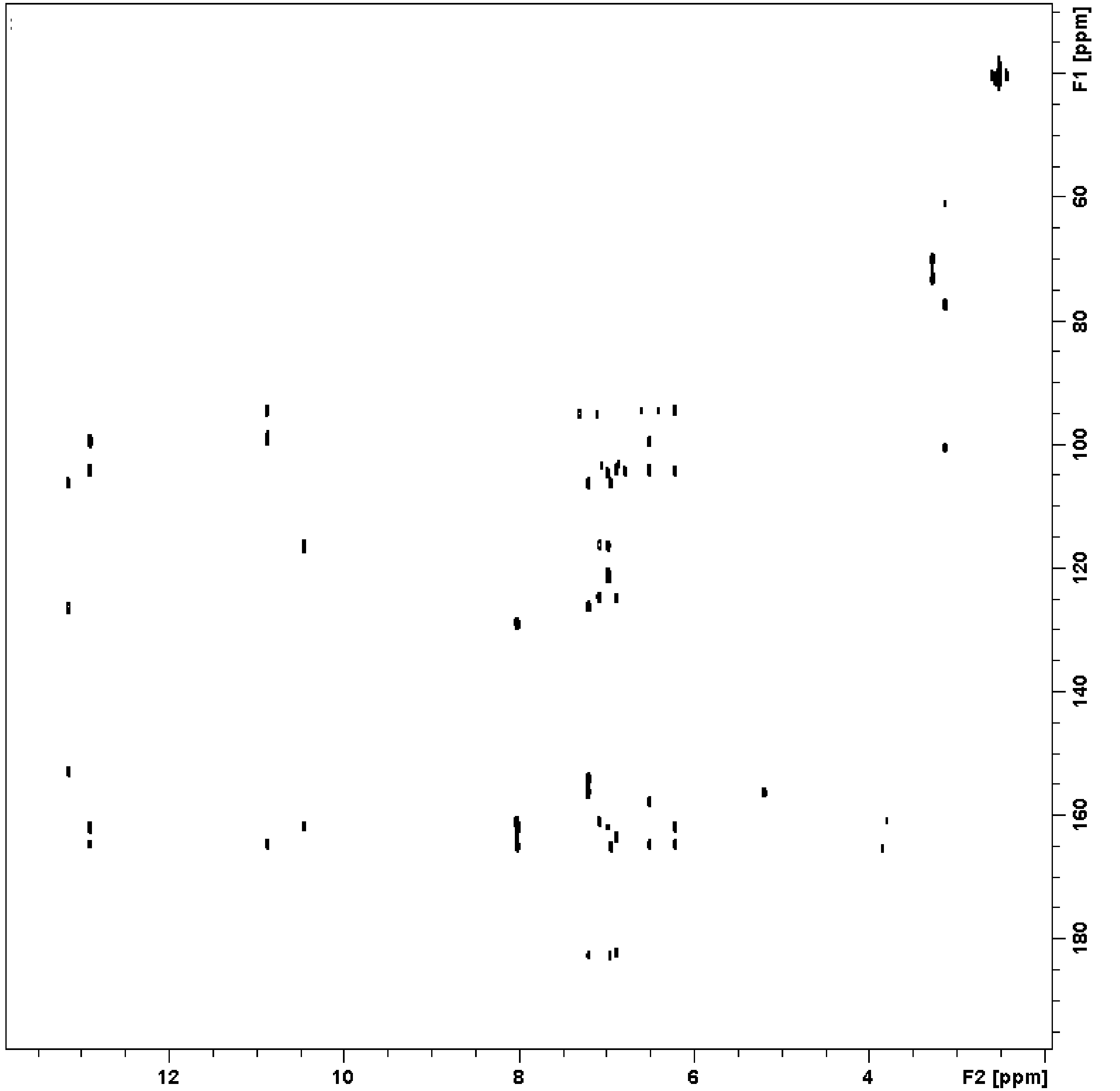

Fig. S4. 2D ^{1}H-^{13}C HSQC NMR spectrum of hinokiflavone 7''-*O*-*β*-glucopyranoside

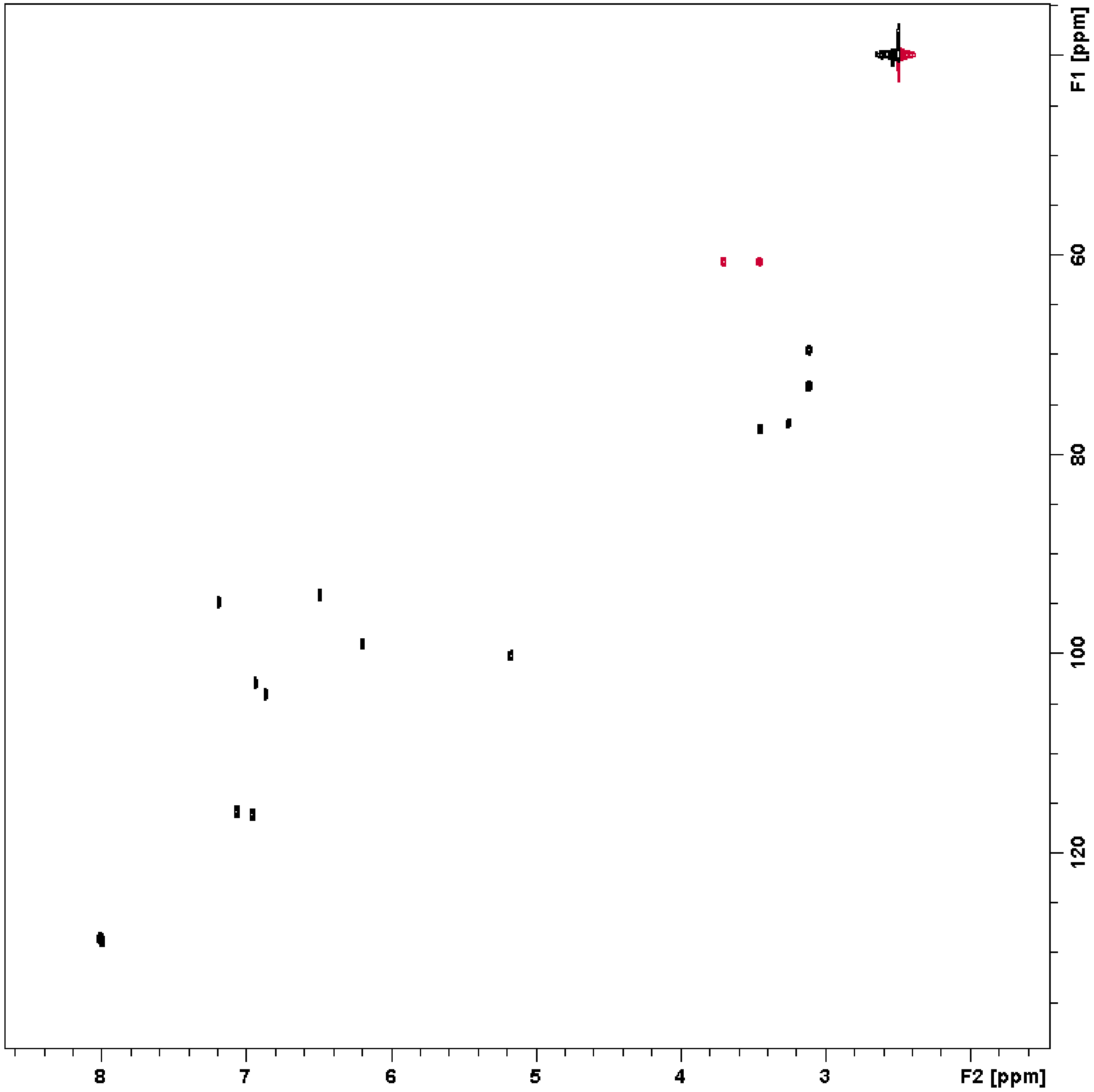

Fig. S5. 2D ^{1}H-^{13}C H2BC NMR spectrum of hinokiflavone 7''-*O*-*β*-glucopyranoside

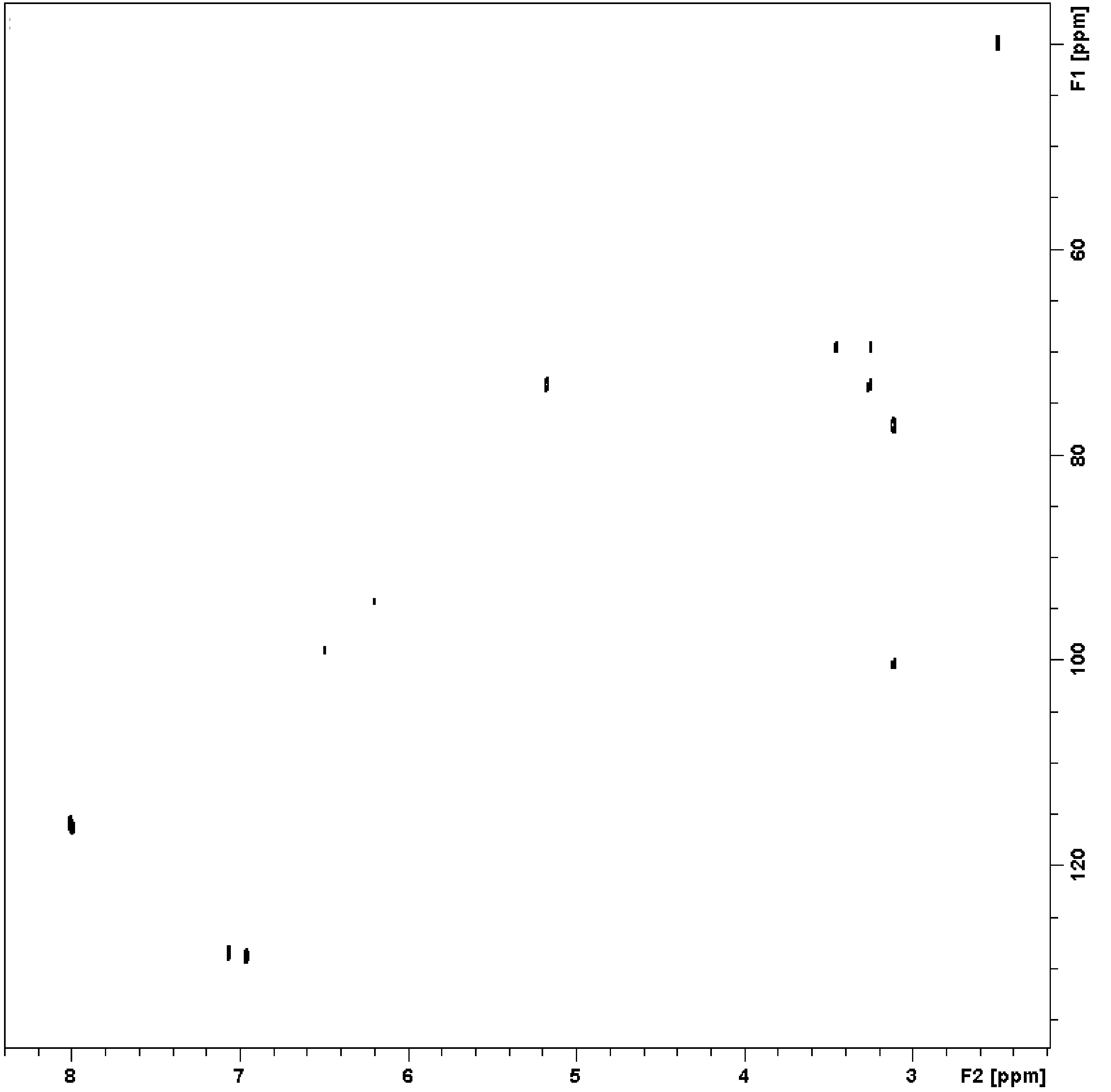

Fig. S6. 2D ^{1}H-^{1}H COSY NMR spectrum of hinokiflavone 7''-*O*-*β*-glucopyranoside

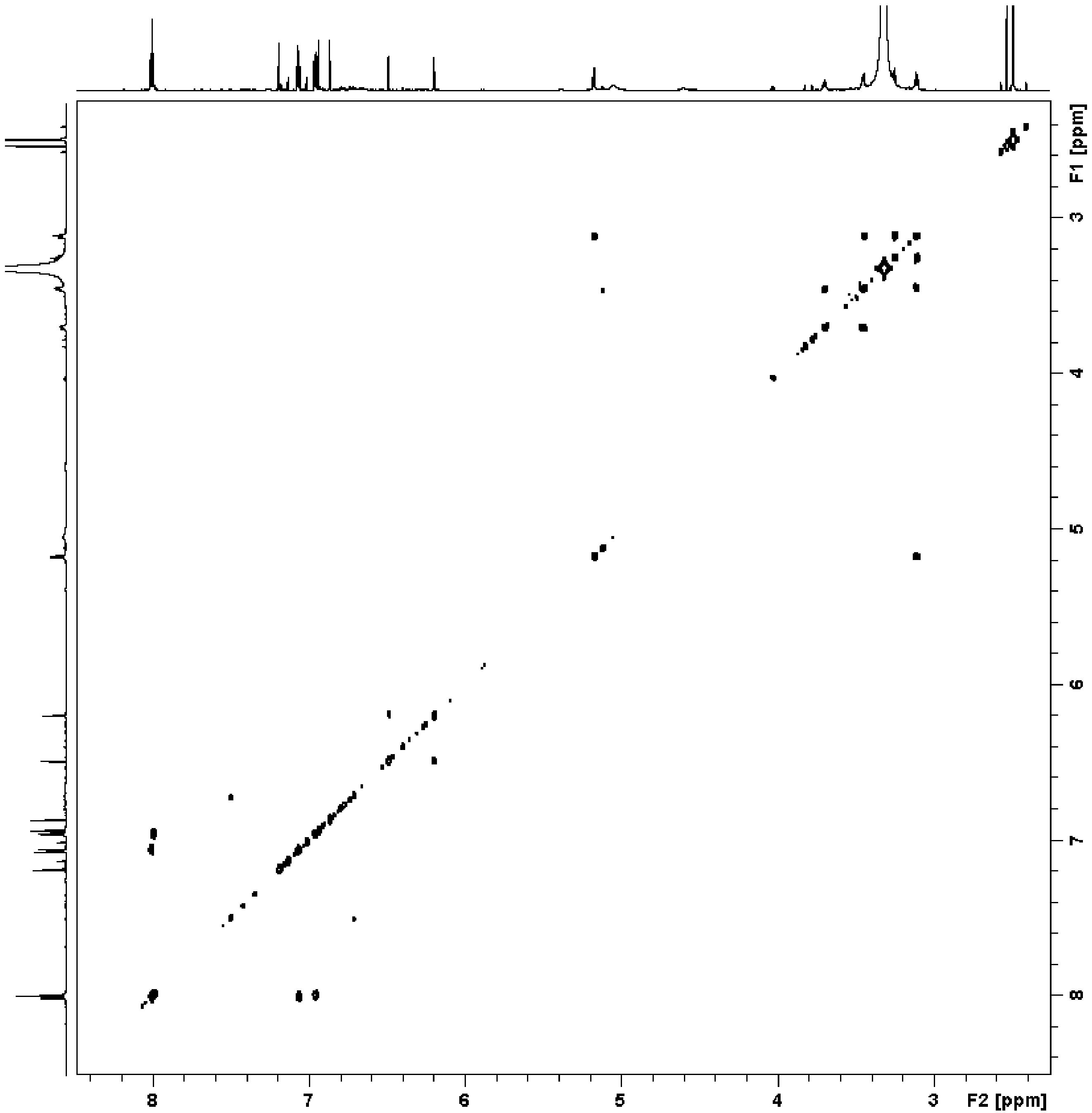

Fig. S7. 2D ^1H-^1H ROESY NMR spectrum of hinokiflavone 7''-*O*-*β*-glucopyranoside

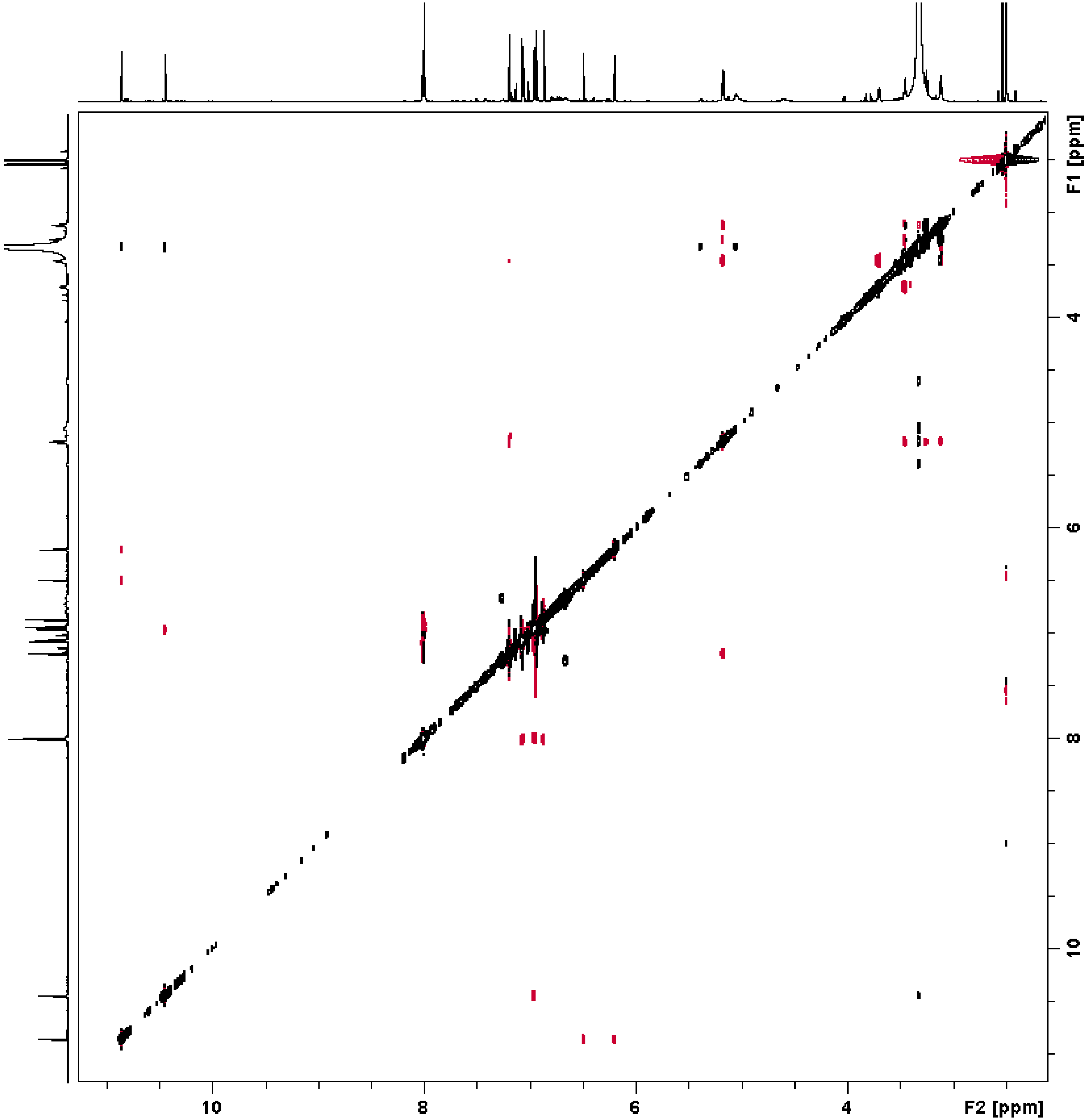

Fig. S8. High-resolution mass spectrum of hinokiflavone 7’’-*O*-*β*-glucopyranoside

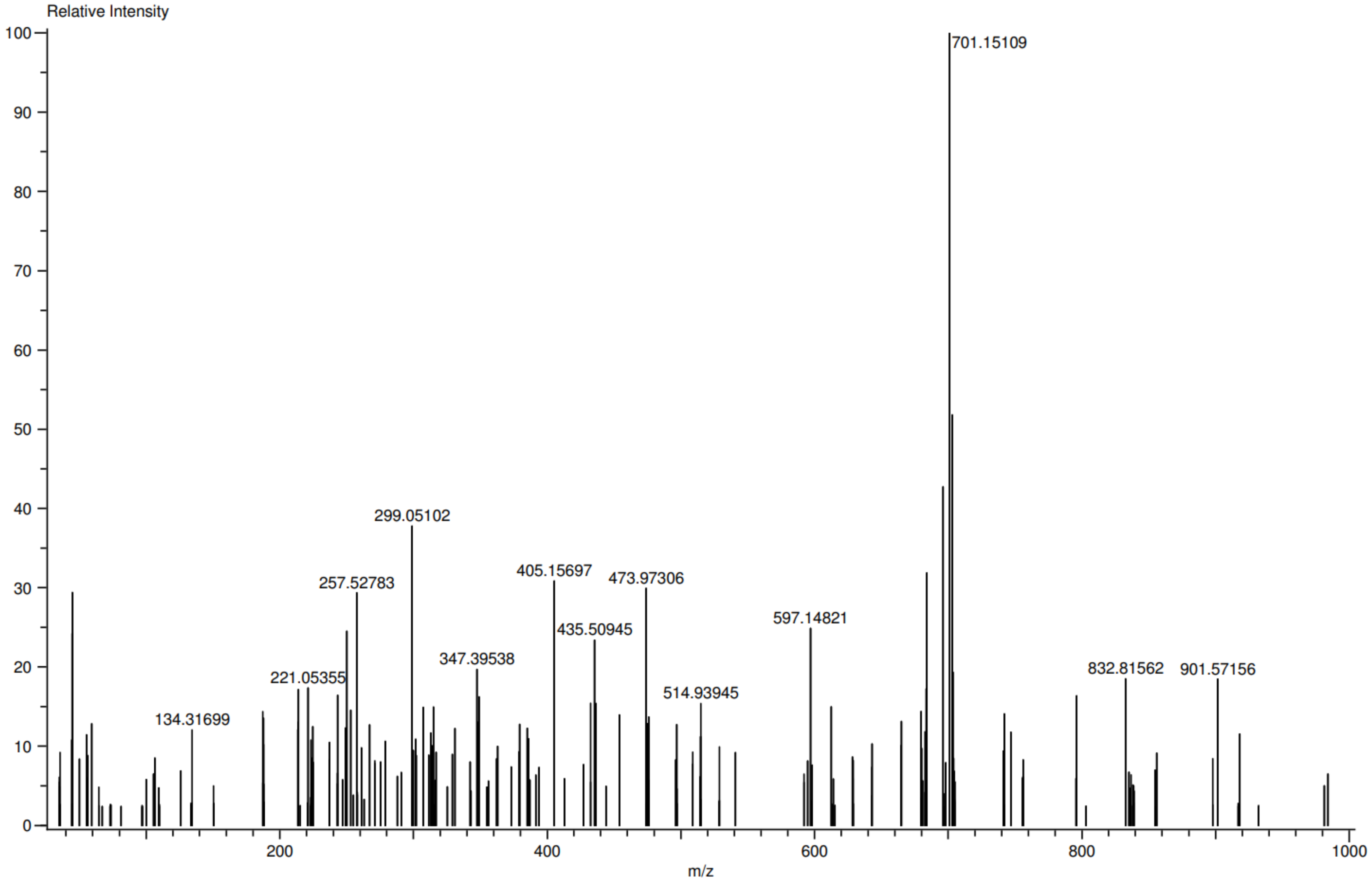

Table S1: ^{1}H and ^{13}C NMR chemical shift values (ppm) and coupling constants (Hz) for quercetin (**1**) in DMSO-D_6 at 298 K.

	**δ ^{1}H**	**δ ^{13}C**
2		146.88
3		135.80
4		175.91
5		160.79
6	6.17 d 2.1	98.25
7		163.97
8	6.39 d 2.1	93.42
9		156.21
10		103.08
1’		122.02
2’	7.66 d 2.3	115.13
3’		145.14
4’		147.78
5’	6.87 d 8.4	115.67
6’	7.53 dd 8.4, 2.3	120.04
3-OH	9.34 s	
5-OH	12.47 s	
7-OH	10.80 s	
3’-OH	9.29 s	
4’-OH	9.59 s	

Table S2: ^1H and ^{13}C NMR chemical shift values (ppm) and coupling constants (Hz) for quercetin 3-*O*-*α*-rhamnopyranoside (**2**) in DMSO-D_6 at 298 K.

	**δ ^1H**	**δ ^{13}C**
2		157.37
3		134.29
4		177.83
5		161.37
6	6.20 d 2.1	98.75
7		164.27
8	6.38 d 2.1	93.69
9		156.52
10		104.15
1’		120.79
2’	7.29 d 2.2	115.72
3’		145.28
4’		148.52
5’	6.85 d 8.3	115.52
6’	7.24 dd 8.3, 2.2	121.18
5-OH	12.65 s	
7-OH	10.87 s	
3’-OH	9.33 s	
4’-OH	9.70 s	
3-*O*-*α*-rhamnopyranoside		
1’’	5.24 d 1.7	101.91
2’’	3.96 dd 3.3, 1.7	70.12
3’’	3.49 dd 9.4, 3.3	70.42
4’’	3.13 t 9.4	71.24
5’’	3.20 dd 9.4, 6.3	70.66
6’’	0.80 d 6.3	17.57

Table S3: ^{1}H and ^{13}C NMR chemical shift values (ppm) and coupling constants (Hz) for quercetin 3-*O*-*β*-galactopyranoside (**3**) in DMSO-D_6 at 298 K.

	**δ ^{1}H**	**δ ^{13}C**
2		156.34
3		133.59
4		177.59
5		161.33
6	6.19 d 2.1	98.77
7		164.24
8	6.40 d 2.1	93.60
9		156.40
10		104.02
1’		121.19
2’	7.52 d 2.3	116.04
3’		144.93
4’		148.57
5’	6.81 d 8.5	115.28
6’	7.66 dd 8.5, 2.3	122.10
5-OH	12.62 s	
7-OH	10.89 s	
3’-OH	9.15 s	
4’-OH	9.73 s	
3-*O*-*β*-galactopyranoside		
1’’	5.37 d 7.7	101.90
2’’	3.56 dd 9.6, 7.7	71.30
3’’	3.36 dd 9.6, 3.4	73.29
4’’	3.64 dd 3.4, 1.1	68.02
5’’	3.32 dt 6.0, 1.1	75.94
6A’’	3.45 dd 10.7, 6.0	60.24
6B’’	3.28 dd 10.7, 6.0	

Table S4: ^{1}H and ^{13}C NMR chemical shift values (ppm) and coupling constants (Hz) for taxifolin 3-O-β-glucopyranoside (**4**) in DMSO-D_6 at 298 K.

	**δ ^{1}H**	**δ ^{13}C**
2	5.45d 7.0	80.77
3	4,81d 7.0	75.43
4		192.86
5		163.55
6	5.87d 2.1	95.99
7		167.19
8	5.90d 2.1	95.07
9		161.81
10		101.25
1’		126.70
2’	6.79d 2.1	114.77
3’		145.24
4’		145.75
5’	6.69d 8.2	115.48
6’	6.67 dd 8.2, 2.1	118.78
5-OH	11.67	
7-OH	10.89	
3’-OH	8.98	
4’-OH	9.06	
3-*O*-*β*-glucopyranoside		
1’’	4.06d 7.6	101.06
2’’	2.94dd 9.4, 7.6	73.57
3’’	2.98dd 9.4, 8.7	76.71
4’’	3.00dd 9.4 8.7	70.01
5’’	2.93 ddd 9.4, 6.2, 2.3	77.27
6A’’	3.60 dd 12.0, 2.3	61.18
6B’’	3.39dd 12.0, 6.2	61.18

Table S5: ^{1}H and ^{13}C NMR chemical shift values (ppm) and coupling constants (Hz) for *taxifolin 7-O-β-glucopyranoside (**5**) in DMSO-D₆ at 298 K.*

	**δ ^{1}H**	**δ ^{13}C**
2	5.05d 11,2Hz	83,42
3	4,57d 11,2Hz	71,88
4		198,73
5		162,93
6	6,16d 2,2Hz	96,90
7		165,58
8	6,13d 2,2Hz	95,56
9		162,56
10		102,23
1’		127,99
2’	6,89d 2,0Hz	115,58
3’		145,14
4’		146,03
5’	6,75d 8,2Hz	115,33
6’	6,77dd 8,2, 2,0 Hz	119,67
7-*O*-*β*-glucopyranoside		
1’’	4,96d 7,7 Hz	99,80
2’’	3,21 dd 8,9, 7,7Hz	73,19
3’’	3,26d 8,9Hz	76,49
4’’	3,14dd 9,8, 8,9Hz	69,67
5’’	3,38 ddd 9,8, 5,8, 2,2Hz	77,25
6A’’	3,66dd 12,0 ,2,2Hz	60,75
6B’’	3,44dd 12,0, 5,8Hz	60,75

Table S6: ^{1}H and ^{13}C NMR chemical shift values (ppm) and coupling constants (Hz) for naringenin (**6**) in DMSO-D_6 at 298 K.

	**δ ^{1}H**	**δ ^{13}C**
2	5.43 dd 12.9, 3.0	78.5
3A	3.26 dd 17.1, 12.9	42.0
3B	2.67 dd 17.1, 3.0	
4		196.5
5		163.6
6	5.87 d 2.2	95.9
7		166.7
8	5.87 d 2.2	95.0
9		163.4
10		101.9
1’		129.0
2’/6’	7.32 ‘d’ 8.6	128.4
3’/5’	6.79 ‘d’ 8.6	115.2
4’		157.8
5-OH	12.13 s	
7-OH	10.77 s	
4’-OH	9.57 s	

Table S7: ^{1}H and ^{13}C NMR chemical shift values (ppm) and coupling constants (Hz) for naringenin 7-*O*-*β*-glucopyranoside (**7**) in DMSO-D_6 at 298 K.

	δ ^{1}H	δ ^{13}C
2	5.49 dd 12.7, 2.9	78.74
3A	3.34 m	42.15
3B	2.73 dd 17.1, 2.9	
4		197.31
5		163.01
6	6.12 d 2.3	96.56
7		165.39
8	6.14 d 2.3	95.51
9		162.83
10		103.32
1’		128.70
2’/6’	7.32 ‘d’ 8.6	128.52
3’/5’	6.79 ‘d’ 8.6	115.25
4’		157.89
5-OH	12.05 s	
4’-OH	9.59 s	
7-*O*-*β*-glucopyranoside		
1’’	4.95 d 7.8	99.67
2’’	3.19 dd 9.0, 7.8	73.09
3’’	3.24 t 9.0	76.38
4’’	3.12 t 9.3	69.55
5’’	3.36 ddd 9.7, 5.7, 2.0	77.15
6A’’	3.64 dd 12.0, 2.0	60.63
6B’’	3.42 dd 12.0, 5.7	
2’’-OH	5.33 s	
3’’-OH	5.08 s	
4’’-OH	5.01 s	
6’’-OH	4.54 s	

Table S8: ^1H and ^{13}C NMR chemical shift values (ppm) and coupling constants (Hz) for eriodictyol 4’-*O*-*β*-glucopyranoside (**8**) in DMSO-D$_6$ at 298 K.

	**δ ^{1}H**	**δ ^{13}C**
2	5.44 dd 12.4, 3.3	78.14
3A	3.20 dd 17.1, 12.4	42.10
3B	2.71 dd 17.1, 3.3	
4		196.21
5		163.53
6	5.87 d 2.2	95.89
7		166.74
8	5.89 d 2.2	95.0 q7
9		162.82
10		101.86
1’		133.32
2’	6.95 d 2.2	114.44
3’		146.83
4’		145.54
5’	7.12 d 8.4	116.63
6’	6.85 dd 8.4, 2.2	117.84
5-OH	12.11 s	
7-OH	10.79 s	
3’-OH	8.71 s	
4’-*O*-*β*-glucopyranoside		
1’’	4.69 d 7.5	102.25
2’’	3.28 dd 9.2, 7.5	73.37
3’’	3.26 dd 9.2, 8.5	75.94
4’’	3.15 dd 9.8, 8.5	69.91
5’’	3.31 ddd 9.8, 6.1, 2.3	77.30
6A’’	3.70 dd 12.0, 2.3	60.84
6B’’	3.46 dd 12.0, 6.1	

Table S9: ^{1}H and ^{13}C NMR chemical shift values (ppm) and coupling constants (Hz) for catechin (**9**) in DMSO-D_6 at 298 K

	δ ^{1}H	δ ^{13}C
2	4.46 d 7.6	81.08
3	3.80 m	66.38
4A	2.64 dd 16.0, 5.4	27.95
4B	2.34 dd 16.0, 8.2	
5		156.24
6	5.87 d 2.3	95.17
7		156.53
8	5.67 d 2.3	93.91
9		155.43
10		99.12
1’		130.67
2’	6.71 d 2.1	114.59
3’		144.91
4’		144.91
5’	6.67 d 8.1	115.14
6’	6.58 dd 8.1, 2.1	118.49
3-OH	4.85 s	
5-OH	9.16 s	
7-OH	8.92 s	
3’-OH	8.84 s	
4’-OH	8.80 s	

Table S10: ^{1}H and ^{13}C NMR chemical shift values (ppm) and coupling constants (Hz) for amentoflavone (**10**) in DMSO-D_6 at 298 K.

	δ ^{1}H	δ ^{13}C
2		163.86
3	6.83 s	103.05
4		181.81
5		161.52
6	6.18 d 2.1	98.91
7		164.20
8	6.46 d 2.1	94.10
9		157.44
10		103.78
1’		121.05
2’	7.99 d 2.5	131.49
3’		120.00
4’		159.57
5’	7.14 d 8.5	116.20
6’	8.00 dd 8.5, 2.5	127.89
5-OH	12.96* s	
7-OH	10.80 s	
4’-OH	10.29 s	
2’’		163.78
3’’	6.79 s	102.66
4’’		182.21
5’’		160.60
6’’	6.40 s	98.65
7’’		161.87
8’’		104.01
9’’		154.55
10’’		103.72
1’’’		121.47
2’’’/6’’’	7.56 ‘d’ 8.9	128.27
3’’’/5’’’	6.71 ‘d’ 8.9	115.83
4’’’		161.11
5’’-OH	13.09* s	
7’’-OH	10.76 s	
4’’’-OH	10.26 s	

*Assignments may be reversed

Table S11: ^{1}H and ^{13}C NMR chemical shift values (ppm) and coupling constants (Hz) for phloretin 2’-*O*-*β*-glucopyranoside (**11**) in DMSO-D_6 at 298 K.

	**δ ^{1}H**	**δ ^{13}C**
1		131.5
2/6	7.02 ‘d’ 8.6	129.3
3/5	6.63 ‘d’ 8.6	115.1
4		155.3
C=O		204.8
αA	3.40 dd 17.3, 7.6	45.0
αB	3.31 m	
β	2.77 t 7.6	29.0
1’		105.2
2’		161.0
3’	6.11 d 2.3	94.4
4’		164.4
5’	5.91 d 2.3	96.8
6’		165.4
4-OH	9.08 s	
4’-OH	10.57 s (br)	
6’-OH	13.49 s	
2’-*O*-*β*-glucopyranoside		
1’’	4.92 d 7.5	100.9
2’’	3.25 ddd 8.9, 7.5, 5.5	73.3
3’’	3.27 dt 8.9, 4.8	76.7
4’’	3.17 ddd 9.7, 8.9, 5.5	69.6
5’’	3.31 m	77.4
6A’’	3.69 ddd 12.1, 5.7, 2.2	60.6
6B’’	3.49 td 12.1, 5.7	
2’’-OH	5.28 d 5.5	
3’’-OH	5.14 d 4.8	
4’’-OH	5.04 d 5.5	
6’’-OH	4.58 t 5.7	

Table S12: ^{1}H and ^{13}C NMR chemical shift values (ppm) and coupling constants (Hz) for roseoside (**12**) in DMSO-D_6 at 298K

	δ ^{1}H	δ ^{13}C
1		41.00
2A	2.41 d 16.8	49.43
2B	2.04 d 16.8	
3		197.42
4	5.77 q 1.3	125.73
5		164.06
6		77.93
7	5.77 d 15.8	130.41
8	5.73 dd 15.8, 6.3	133.39
9	4.31 p 6.3	74.67
10	1.17 d 6.3	20.92
11	0.91 s	24.13
12	0.92 s	23.10
13	1.80 d 1.3	18.96
9-*O*-*β*-glucopyranoside		
1’’	4.16 d 7.8	100.96
2’’	2.92 dd 8.9, 7.8	73.76
3’’	3.10 t 8.9	76.91
4’’	3.02 m	70.11
5’’	3.02 m	76.87
6A’’	3.63 dd 11.6, 1.8	61.17
6B’’	3.40 dd 11.6, 5.5	

Table S13: ^{1}H and ^{13}C NMR chemical shift values (ppm) and coupling constants (Hz) for chlorogenic acid (**13**) and methyl chlorogenate (**13m**) in DMSO-D_6 ved 298K

	Chlorogenic acid		Methyl chlorogenate	
	**δ ^{1}H**	**δ ^{13}C**	**δ ^{1}H**	**δ ^{13}C**
1		73,96		73,58
2A	2,11 m	37,70	2,04 m	37,65
2B	1,77 m		1,80 m	
3	3,95 m	68,57	3,90 m	67,42
4	3,58 dd 10.1, 7.6	70,87	3,59 dd 9.0, 6.5	69,87
5	5,04 dd 10.1, 5.4	71,47	5,09 dd 10.8, 6.5	71,32
6A	2,02 m	36,71	2.12m	35,65
6B	1,96m		1,95m	
7		174,10		175,40
1’		125,86		126,08
2’	7,05 d 2.17	115,24	7,04d 2.17	115,09
3’		146,01		146,09
4’		148,95		148,79
5’	6,78d 8.4	116,33	6,78d 8.4	116,21
6’	6,98 dd 8.4, 2.0	121,84	6,99 dd 8.4, 1.9	121,82
7’	7,40 d 16.0	145,60	7,44 d 15.8	145,42
8’	6,12d 16.0	114,77	6,16d 15.8	114,36
9’		165,86		166,21
3-OH	9,16s			
4-OH	9,57s			
7-OCH3			3,57s	52,27
7-COOH	11,80s			

Table S14: ^1H and ^{13}C NMR chemical shift values (ppm) and coupling constants (Hz) for two rotamers of catechin-(7,8-bc)-4-(3,4-dihydroxyphenyl)-dihydro-2(3H)-pyranone (**14**) in DMSO-D$_6$ at 298 K, where signals of minor rotamer are shown in red.

	δ ^{1}H	δ ^{13}C
2	4.57 d 7.6	81.37
	4.62 d 7.2	81.08
3	3.94m	65.71
	3.93m	65.66
4A	2.82 dd 16.2, 5.3	27.49
	2.75 dd 16.2, 5.2	27.20
4B	2.48 dd 16.2, 8.0	27.49
	2.54 dd 16.2, 7.9	27.20
5		154.04
		153.95
6	6.165 s	98.36
	6.171 s	98.31
7		153.19
		153.21
8		105.57
		105.48
9		150.19
		150.26
10		100.08
		100.00
1’		130.13
		130.17
2’	6.75 d 2.1	114.59
	6.74 d 2.1	114.52
3’		144.99
4’		145.17
5’	6.72 d 8.2	115.24
	6.71 d 8.2	115.25
6’	6.61 m	118.45
	6.60 m	118.35
3-OH	5.04 d 5.1	
	5.07 d 4.9	
5-OH	9.67 s	
	9.66 s	
3’-OH	8.877 s	
	8.876 s	
4’-OH	8.90 s	
	8.89 s	
1’’		133.06
		133.05
2’’	6.47 d 2.3	114.18
	6.46 d 2.3	114.21
3’’		145.09
		145.05
4’’		144.10
5’’	6.63 m	115.63
	6.62 m	115.65
6’’	6.37 m	117.46
	6.36 m	117.45
7’’	4.30	33.30
	4.29	33.24
8A’’	3.11	37.17
	3.09	37.06
8B”	2.75	37.17
	2.75	37.06
9’’		167.91
		167.88
3’’-OH	8.824 s	
	8.817 s	
4’’-OH	8.74 s	
	8.73 s	

Table S15: ^{1}H and ^{13}C NMR chemical shift values (ppm) and coupling constants (Hz) for Mururin A (**16**) in DMSO-D$_6$ at 298 K

	δ ^{1}H	δ ^{13}C
2	4.89 d 6.4	81.74
3	4.06 ddd 7.0, 6.4, 4.8	64.82
4A	2.79 dd 16.2, 4.8	25.82
4B	2.68 dd 16.2, 7.0	
5		151.77
6		99.67
7		148.71
8	6.66 s	97.21
9		157.92
10		103.50
1’		129.48
2’	6.71 d 2.1	114.12
3’		145.14
4’		145.23
5’	6.70 d 8.2	115.41
6’	6.60 dd 8.2, 2.1	118.01
3-OH	5.26 d 4.4	
1’’		106.22
2’’		146.62
3’’	6.75 s	103.33
4’’		143.74
5’’		152.40
6’’	7.34 s	109.24
7’’		141.58
8’’	6.09 s	92.07
9’’		161.16
